# Frequency modulation increases the specificity of time-resolved connectivity: A resting-state fMRI study

**DOI:** 10.1162/netn_a_00372

**Published:** 2024-10-01

**Authors:** Ashkan Faghiri, Kun Yang, Andreia Faria, Koko Ishizuka, Akira Sawa, Tülay Adali, Vince Calhoun

**Affiliations:** Tri-Institutional Center for Translational Research in Neuroimaging and Data Science (TReNDS), Georgia State University, Georgia Institute of Technology, and Emory University, Atlanta, GA, USA; Department of Psychiatry, Johns Hopkins University School of Medicine, Baltimore, MD, USA; Department of Radiology and Radiological Science, Johns Hopkins University School of Medicine, Baltimore, MD, USA; Departments of Psychiatry, Neuroscience, Biomedical Engineering, Genetic Medicine, and Pharmacology, Johns Hopkins University School of Medicine, Baltimore, MD; Department of Mental Health, Johns Hopkins Bloomberg School of Public Health, Baltimore, MD; Deptartment of CSEE, University of Maryland, Baltimore County, Baltimore, MD, USA; School of Electrical and Computer Engineering, Georgia Institute of Technology, Atlanta, GA, USA

**Keywords:** Resting-state fMRI, Sliding window Pearson correlation (SWPC), First episode of psychosis (FEP), Frequency modulation, Independent component analysis, Single sideband modulation (SSB)

## Abstract

Representing data using time-resolved networks is valuable for analyzing functional data of the human brain. One commonly used method for constructing time-resolved networks from data is sliding window Pearson correlation (SWPC). One major limitation of SWPC is that it applies a high-pass filter to the activity time series. Therefore, if we select a short window (desirable to estimate rapid changes in connectivity), we will remove important low-frequency information. Here, we propose an approach based on single sideband modulation (SSB) in communication theory. This allows us to select shorter windows to capture rapid changes in the time-resolved functional network connectivity (trFNC). We use simulation and real resting-state functional magnetic resonance imaging (fMRI) data to demonstrate the superior performance of SSB+SWPC compared to SWPC. We also compare the recurring trFNC patterns between individuals with the first episode of psychosis (FEP) and typical controls (TC) and show that FEPs stay more in states that show weaker connectivity across the whole brain. A result exclusive to SSB+SWPC is that TCs stay more in a state with negative connectivity between subcortical and cortical regions. Based on all the results, we argue that SSB+SWPC is more sensitive for capturing temporal variation in trFNC.

## INTRODUCTION

The human brain is a highly dynamic and complex system, and our measurements of this organ can capture many aspects of this system. In recent years, network science tools have been used extensively for this purpose with much success in capturing both the complexity (Bassett & Gazzaniga, 2011) and the dynamism (Deco et al., 2011) of the human brain. Typically, to use network science tools, one must first construct the network itself from the recorded brain data. We can have many different types of networks depending on the data and the network construction step. We can use functional data like functional magnetic resonance imaging (fMRI) to capture blood oxygenation level–dependent (BOLD) signals and construct functional time-resolved networks. The step of going from the data to the network is quite challenging as we often do not have direct measurements of the network, and there are many open questions about network reconstruction for functional brain networks (Erhardt et al., 2011; Korhonen et al., 2021). A network consists of a set of nodes and the edges that connect these nodes together. To estimate the nodes from the data, we can use methods that are based on prior works and atlases (Biswal et al., 1995; Fox et al., 2006) or use data-driven approaches like independent component analysis (ICA) (Calhoun et al., 2009; Faghiri et al., 2018). Some methods, such as constrained ICA (Du et al., 2017; Lin et al., 2010), blend these two categories. The other element of networks are the edges between different nodes, which in the neuroimaging field are often referred to as connectivity (e.g., functional connectivity in the case of functional networks).

It is common to use the term functional network connectivity (FNC) when the nodes themselves are a type of network. For example, if we use ICA to estimate the nodes, these nodes (often called intrinsic connectivity networks or components) are essentially networks themselves (Erhardt et al., 2011). In this work, we use the term FNC exclusively, but the proposed method can also be used to estimate FC. If our aim is to capture the dynamics of the human brain, we should estimate FNC in a manner that is temporally resolved (time-resolved functional network connectivity). Many methods have been proposed to estimate time-resolved functional network connectivity (trFNC) from nodes’ time series. The most well-known method for estimating trFNC is the sliding window Pearson correlation (SWPC), which essentially pairs a sliding window with a sample Pearson correlation (Allen et al., 2014). Another method replaces the sliding window part of SWPC with a filter bank to capture trFNC across all of its spectrum (Faghiri et al., 2022b). Some methods aim to estimate trFNC in a more instantaneous fashion, like instantaneous phase synchrony (Honari et al., 2021; Pedersen et al., 2017), the multiplication of temporal derivative (Shine et al., 2015), or instantaneous shared trajectory (Faghiri et al., 2020). Some methods do not estimate trFNC directly but aim to model it at different levels, like methods based on hidden Markov modeling (Vidaurre et al., 2017). Interested readers can check the many papers that have reviewed the many existing methods for time-resolved connectivity estimation (Iraji et al., 2021; Lurie et al., 2020).

As mentioned above, SWPC is a widely used method likely because of both its simplicity and the fact that the Pearson correlation itself has been studied for many years (Rodgers & Nicewander, 1988). Looking at SWPC as a system with its subsystem, we can see that it consists of a high-pass filter that is applied to the inputs (nodes time series) in addition to a coupling function and low-pass filter (Faghiri et al., 2022b). The window size and window shape selection in SWPC are equivalent to designing these two filters. By selecting smaller window sizes, the high-pass filter cutoff frequency is increased, meaning that we will remove more low-frequency information in the sample space (or activity domain). This can be problematic as the low-frequency content of resting-state fMRI (rsfMRI) is quite important (Lee et al., 2013). To solve this issue, some works have suggested a lower bound on the window size for SWPC. For example, Leonardi and Van De Ville (2015) have suggested that assuming that resting-state fMRI data has a lower frequency bound of 0.01 Hz, we need to select window sizes larger than 100 seconds to ensure that the high-pass filter in SWPC does not remove important low-frequency information. This statement is accurate but with an important caveat. The window size in SWPC also affects its low-pass filter, which is applied in the connectivity domain. If we choose a large window size, the low-pass filter’s cutoff decreases, meaning we might smooth out important information in the connectivity time series. It is important to note that we do not have a direct measurement of the connectivity time series; instead, we estimate this time series from the activity time series. So, while there are many studies on the frequency profile of activity time series (Biswal et al., 1995; Chen & Glover, 2015; Fransson, 2005; Trapp et al., 2018), it is quite challenging to discuss the connectivity frequency profile. The challenging part is that we cannot measure connectivity directly, and we have to rely on estimators with their own transfer function that would impact the spectral properties of the connectivity (Faghiri et al., 2022b). This is a circular problem: we need to examine the frequency profile of the connectivity time series for selecting the window of the SWPC method, but to get the frequency profile of connectivity, we need to use SWPC with a specific window, which in turn impacts the estimated frequency profile.

In this work, inspired by a modulation scheme in communication theory, we propose a method that allows us to select window size for SWPC without worrying about removing important low-frequency information from the rsfMRI data (Faghiri et al., 2022a). We evaluate the proposed method using both simulated and real data in addition to using it to analyze a rsfMRI dataset.

## METHODS

### Sliding Window Pearson Correlation

Sliding window Pearson correlation (SWPC) is an extension of the classical Pearson correlation estimator, where we can capture the correlation coefficients of different temporal segments using a sliding window. The SWPC can be formulated as:

$$r_{x,y}\left(t;\Delta \right)=\sum_{\tau =t-\Delta }^{t+\Delta }\frac{\left(x\left(\tau \right)-{\hat{\mu }}_{x}\left(t\right)\right)\left(y\left(\tau \right)-{\hat{\mu }}_{y}\left(t\right)\right)}{{\hat{\sigma }}_{x}\left(t\right){\hat{\sigma }}_{y}\left(t\right)}$$ (1)

where $\hat{\mu }$_*x*_(*t*) and $\hat{\mu }$_*y*_(*t*) are average of *x*(*t*) and *y*(*t*) for *t* ∈ [*t* − Δ, t + Δ], while $\hat{\sigma }$_*x*_(*t*) and $\hat{\sigma }$_*y*_(*t*) are standard deviation of *x*(*t*) and *y*(*t*), respectively, for the range *t* ∈ [*t* − Δ, t + Δ]. Note that Δ is the free parameter for Equation 1, which is why it is written after a semicolon.

As discussed in our previous work (Faghiri et al., 2022b), the SWPC can be broken into three subsystems. The first subsystem, which both inputs pass through, is the part where the moving average is subtracted from the data (i.e., *x*(*τ*) − $\hat{\mu }$_*x*_(*t*) in Equation 1, where $\hat{\mu }$_*x*_(*t*) is the moving average of *x*(*t*)). It is trivial to show that removing the moving average of a time series from itself is equivalent to passing the time series (i.e., *x*(*t*) and *y*(*t*)) through a high-pass filter. The second subsystem is a coupling subsystem, which multiplies the outputs of the previous subsystem. The final subsystem is a low-pass filter applied to the coupled time series. Based on this view, the design of the window (its shape and length) is essentially a filter design step for the two filtering subsystems. As mentioned previously, one of the shortcomings of SWPC arises because of the first high-pass filter of this method (i.e., *x*(*τ*) − $\hat{\mu }$_*x*_(*t*)). If the window selected for SWPC is small, low-frequency portions of the signals *x*(*t*) and *y*(*t*) are filtered out, which in turn can possibly worsen the estimation of the time-resolved correlation. By selecting large window sizes, we can remedy this issue, but the side effect of such a choice is that we might smooth out important high-frequency time-resolved connectivity information. In this work, we propose a method that allows us to select smaller window sizes for SWPC without removing low-frequency contents of *x*(*t*) and *y*(*t*) by adding an additional step to the classic SWPC method.

### Single Sideband Modulation + Sliding Window Pearson Correlation

**Figure 1.**
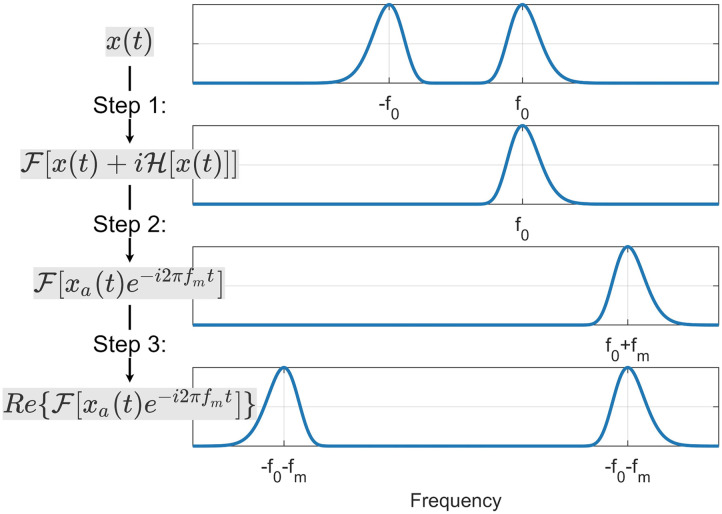
Illustration of the effect of the single sideband modulation method on the spectrum of the signal. This method has three main steps. In the first step, the analytic signal is calculated using the Hilbert transform. For the second step, the spectrum of the analytic signal is modulated to the right (toward the positive frequencies). In the third step, the negative sideband is added back to the signal by calculating the real part of the modulated signal resulting from the previous step.

Single sideband modulation (SSB) is a method that can modulate the frequency content of a time series while keeping the signal real. This method can be broken into three different steps. First, the analytic signal is calculated using the Hilbert transform. Second, this complex signal is multiplied with a complex exponential term. Finally, for the third step, the real part of the result of the previous step is calculated. The mathematical formulation of these three steps for *x*(*t*) can be simply written as

$$x^{ssb}\left(t;f_{m}\right)=𝓡𝓮\left\{x_{a}\left(t\right)e^{-j2\pi f_{m}t}\right\}$$ (2)

where *f*_*m*_ is the modulation frequency, and *x*_*a*_(*t*) is the analytic signal calculated using the Hilbert transform *x*_*a*_(*t*) = *x*(*t*) + *j*𝓗{ *x*(*t*)}. Figure 1 demonstrates the steps of SSB in the frequency domain.

**Figure 2.**
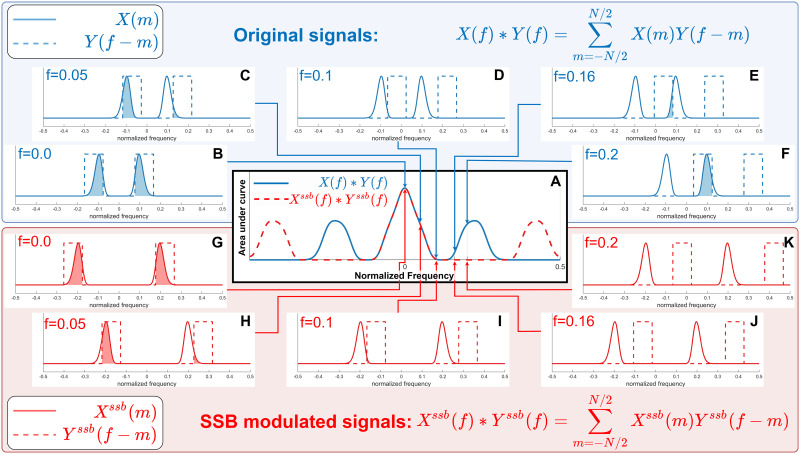
A visual demonstration of why the proposed SSB+SWPC method works. As we saw in the SWPC formula (Equation 1), the coupling part of SWPC is essentially a multiplication in the time domain, which translates into a convolution in the frequency domain. Convolution can be viewed as a two step operator: shifting one signal (the dashed lines in this figure) while keeping the other one fixed (the solid lines in this figure) and calculating the area under the curve of the multiplication of these two signals. Therefore, as long as the area under the curve for two convolutions is the same, the convolution results are equal for the two operators. Here, we see that the area under the curve of the original signal (blue lines) and SSB modulated signals (red lines) are the same as long as the negative sideband of the shifted signal does not overlap with the positive sideband of the fixed line. That is, for frequencies below 0.1 Hz, the original convolution results (panels B, C, and D) are the same as the SSB modulated signal convolution (panels G, H, and I). The two convolutions diverge for shift values higher than 0.1 Hz (subfigures E and F for the original signals and J and K for the SSB modulated signals).

By modulating the frequency content of the signal pairs *x*(*t*) and *y*(*t*) before estimating the SWPC, we can select small window sizes without worrying about filtering out low-frequency information of *x*(*t*) and *y*(*t*). The formula for this method, which we call SSB+SWPC, is:

$$r_{x,y}^{ssb}\left(t;\Delta ,f_{m}\right)=\sum_{\tau =t-\Delta }^{t+\Delta }\frac{\left(x^{ssb}\left(\tau ;f_{m}\right)-{\hat{\mu }}_{x}^{ssb}\left(t\right)\right)\left(y^{ssb}\left(\tau ;f_{m}\right)-{\hat{\mu }}_{y}^{ssb}\left(t\right)\right)}{{\hat{\sigma }}_{x}^{ssb}\left(t\right){\hat{\sigma }}_{y}^{ssb}\left(t\right)}$$ (3)

where ${\hat{\mu }}_{x}^{ssb}$(*t*) and ${\hat{\sigma }}_{x}^{ssb}$ (*t*) are the moving average and moving standard deviation of the modulated signal *x*^*ssb*^(*t*; *f*_*m*_). Note that Δ and *f*_*m*_ are the free parameters of the method and are selected by the user. The reason we can use SSB to modulate *x*(*t*) and *y*(*t*) before estimating the SWPC is because of the spectral property of the coupling subfunction of SWPC. As mentioned before, this subfunction is essentially a multiplication in the time domain (i.e., (*x*(*τ*) − $\hat{\mu }$_*x*_(*t*))(*y*(*τ*) − $\hat{\mu }$_*y*_(*t*)) in Equation 1). Multiplication in the time domain is equivalent to circular convolution in the frequency domain (if the discrete Fourier transform is used for the transformation). Circular convolution can be summarized as flipping and shifting one signal with a specific value and then calculating the area under the curve of the multiplication of the shifted and fixed signal (see Figure 2). We can ignore the flipping step as the spectrums are symmetric. If we modulate *x*(*t*) and *y*(*t*) using the SSB modulation method, the upper and lower sideband of these signals’ Fourier transforms (*X*^*ssb*^(*f*; *f*_*m*_) and *Y*^*ssb*^(*f*; *f*_*m*_), respectively) are shifted opposite to each other to make sure the spectrum remains symmetric (hence why the modulation results are real values). Then, the sum of the area under the curve of *X*^*ssb*^(*f*; *f*_*m*_) × *Y*^*ssb*^(*f* − *f*_0_; *f*_*m*_) (*f*_0_ is the frequency shift done as a part of circular convolution) is the same as the area under the curve of *X*(*f*) × *Y*(*f* − *f*_0_) for specific shift frequencies *f*_0_. This can be seen in Figure 2 for specific *f*_0_ values (panels B, C, and D for *Y*(*f* − *f*_0_) and G, H, and I for *Y*^*ssb*^(*f* − *f*_0_; *f*_*m*_) in Figure 2). But when the shift value is high enough so that the negative sideband of *Y*^*ssb*^(*f* − *f*_0_; *f*_*m*_) starts overlapping with the positive sideband of *X*^*ssb*^(*f*; *f*_*m*_), the result of the two convolutions will diverge (see Figure 2A). This value depends on the lowest frequency of the two signals (i.e., *X*(*f*) and *Y*(*f*)), meaning that if the two signals have no values at frequencies below *a* > 0, for shift values lower than 2*a*, *X*(*f*) ⊛ *Y*(*f*) and *X*^*ssb*^(*f*) ⊛ *Y*^*ssb*^(*f*) are the same (⊛ is the operator symbol for circular convolution). Contrary to what one might think, this does not mean that *X*^*ssb*^(*f*) ⊛ *Y*^*ssb*^(*f*) has worse estimation for *f* > 2*a*, rather, it just means that for these frequencies, *X*(*f*) ⊛ *Y*(*f*) ≠ *X*^*ssb*^(*f*) ⊛ *Y*^*ssb*^(*f*). As we can see in Figure 2A, the second bump of *X*(*f*) ⊛ *Y*(*f*) is the result of overlap of positive sideband of *X*(*f*) with negative sideband of shifted version of *Y*(*f*).

To summarize, SSB+SWPC (the proposed method) adds a modulation step to the classic SWPC method, which allows us to select small window sizes for SWPC without worrying about the lower frequency bounds on the activity time series.

### Method Evaluation Using Synthesized Data

To evaluate the performance of the proposed method, we designed two sets of simulations. These first simulations are designed to evaluate if SSB+SWPC improves time-resolved Pearson correlation. The second one is designed to compare a pipeline that uses SSB+SWPC and one that uses classic SWPC in their performance regarding the estimation of states. In both simulation scenarios, the sampling frequency was set to 2 Hz, and each simulation iteration was repeated 1,000 times. Additionally, the activity signals were band-limited (between 0 and 0.1 Hz) in both scenarios.

We first generated two random time series for the first set of simulations. Next, we filtered these time series to have a specific bandwidth. For this filtration, we used a Chebyshev Type 2 low-pass filter (with the lowest possible order to achieve a maximum of 3 decibels attenuation in the passband and a minimum of 30 decibels attenuation in the stopband). Assume we call these filtered time series *x*(*t*) and *y*(*t*). Next, we generated a connectivity time series to have a specific frequency by using the cosine function *C*(*t*) = 0.7cos(2*πf*_corr_*t*). For this simulation, it was assumed that variance is constant throughout time and is equal to one. In this case, the covariance matrix is:

$$\Sigma \left(t\right)=\left[\begin{array}{cc} 1 & 0.7\cos\left(2\pi f_{\text{corr}}t\right)\\ 0.7\cos\left(2\pi f_{\text{corr}}t\right) & 1 \end{array}\right]$$ (4)

Next, we used Cholesky decomposition to project the two random uncorrelated time series *x*(*t*) and *y*(*t*), into new time series that are correlated. In other words, we first decomposed the covariance matrix Σ(*t*) at each time point using Cholesky decomposition **Σ**(*t*) = ***U***(*t*)^*T*^***U***(*t*) where ***U***(*t*) is an upper triangular matrix. By multiplying ***U***(*t*) with the vector [*x*(*t*), *y*(*t*)] we will have a new time series [*x*_c_(*t*), *y*_c_(*t*)] that are correlated at each time point according to *C*(*t*) = 0.7cos(2*πf*_corr_*t*):

$$\left[x_{\text{c}}\left(t\right),y_{\text{c}}\left(t\right)\right]=\left[x\left(t\right),y\left(t\right)\right]U\left(t\right)$$ (5)

We then used both the proposed method, SSB+SWPC, and the vanilla SWPC approach to estimate time-resolved Pearson correlation (i.e., $\tilde{C}$ (*t*)). To evaluate the performance of the two estimators, we used both sample Pearson correlation and root-mean-square error (RMSE) between the estimated and the true value. Although the mean-square error is more commonly used when evaluating the performance of estimators, in our specific case (constructing whole-brain networks), we care more about the pattern of connections rather than their actual values; therefore, we include both evaluation metrics.

This simulation has four free parameters. There are two parameters for the data generation phase, namely the cutoff frequency of the Chebyshev low-pass filter (which determines the true bandwidth of the time series) and *f*_*corr*_ (which determines how fast connectivity changes). There are also two free parameters for the analysis: the window sizes for both SSB+SWPC and SWPC methods and the modulation frequency used for SSB. As mentioned above, the maximum value of the true correlation is set to 0.7, which can be argued to be high compared to real data; note that this is the maximum value and not the average correlation, so it is not that unlikely to happen in real data. To answer this argument, we repeated this scenario with 0.3 as the maximum value for correlation. Additionally, we explored what would happen if the correlation is always equal to zero, that is, does not change with time. For this last case, the evaluation metric was RMSE as we cannot calculate sample Pearson correlation when one of the time series is constant.

For the second set of simulations, our goal was to evaluate the performance of SSB+SWPC when it is used in a pipeline to estimate connectivity states of the data, assuming we know the true number of the states. This pipeline is essentially the same pipeline we use to estimate the connectivity states of the fMRI data later. We first start by generating six random uncorrelated time series (each with 1,000 time points). Then, we filter them using low-pass Chebyshev Type 2, similar to the previous simulation. Next, we generate two six by six matrices to act as the connectivity states. For the first state, the first three time series are highly correlated, while the correlation is zero elsewhere. The last three time series are connected for the second state, while the correlation is zero elsewhere. The correlation amplitude is 0.7 for all connected time series. In other words, the covariance matrix for the two states is:

$$\Sigma_{1}=\left[\begin{array}{cccccc} 1 & 0.7 & 0.7 & 0 & 0 & 0\\ 0.7 & 1 & 0.7 & 0 & 0 & 0\\ 0.7 & 0.7 & 1 & 0 & 0 & 0\\ 0 & 0 & 0 & 1 & 0 & 0\\ 0 & 0 & 0 & 0 & 1 & 0\\ 0 & 0 & 0 & 0 & 0 & 1 \end{array}\right],\;\Sigma_{2}=\left[\begin{array}{cccccc} 1 & 0 & 0 & 0 & 0 & 0\\ 0 & 1 & 0 & 0 & 0 & 0\\ 0 & 0 & 1 & 0 & 0 & 0\\ 0 & 0 & 0 & 1 & 0.7 & 0.7\\ 0 & 0 & 0 & 0.7 & 1 & 0.7\\ 0.7 & 0 & 0 & 0 & 0.7 & 1 \end{array}\right]$$

Next, we partition the uncorrelated time series into segments based on the state length. For example, if the state length is 10 samples, we have 10 time points in state one, followed by 10 time points in state two periodically until we have segmented the whole temporal length of the time series. For each temporal segment, we multiply the six time series by the Cholesky decomposition of the two states’ covariance matrix. Therefore, in that specific segment, we have time series that have correlation matrices depending on the state of the mentioned state. To estimate these correlation matrices, we first use both SSB+SWPC and SWPC methods to estimate connectivity between the six generated time series. Next, we concatenate all the estimated connectivity values into a big matrix (for each method separately) with a size equal to *T*′ by 15 (the number of unique connectivity values at each window, which is 6 × 5/2) where *T*′ = 1,000 − (*window size*) + 1. We used *k*-means clustering to cluster the estimated connectivity matrices into two clusters (i.e., we assume we know the true number of states for this simulation). We use *k*-means implemented in MATLAB software, which uses the *k*-means++ algorithm for centroid initialization (Arthur & Vassilvitskii, 2007). The clustering was repeated 20 times, with the maximum number of iterations set to 500. To evaluate the performance of the two pipelines, we calculated the correlation between the estimated cluster centroid and the associated true state connectivity matrix. There are three free parameters for this simulation. There is the true state length, window size (for calculating SSB+SWPC and SWPC), and modulation frequency (only for SSB+SWPC). Technically, there is also the filter cutoff frequency, which can be changed, but in this work, we have kept that parameter constant.

We also wanted to discuss the impact of using the Cholesky decomposition approach mentioned above on the spectrum of the activity signals. It is very clear that the spectrum changes by multiplying a time series by another time series (output of the Cholesky decomposition). As this second signal comes from a sinusoidal signal, it has a very narrow frequency content and has little impact on the spectrum of the activity signal. Therefore, we argue that we can ignore this change in the spectrum of the activity signal.

### Method Evaluation Using Real Data

Using simulated or synthesized data enables us to evaluate the performance of novel methods in a controlled way. Still, testing the method’s performance using real data is also quite beneficial. However, this is often quite challenging, especially for data that is the focus of this paper, namely, the resting-state fMRI dataset. To evaluate the performance of any estimator, we need to know the ground truth, which we often do not have. In addition, it is unclear how best to compare the results of a novel method with an older method for evaluation, as we do not know what a “good” result looks like. Note that we believe that the goal of a good estimator should be to provide an estimation as close as possible to the ground truth, not to provide estimations that can result in better comparison results between group A and group B using the estimated values. And this is why evaluation using real data is challenging for resting-state fMRI connectivity studies. We often do not know what a good network looks like. Here, we develop a metric that we believe can be used to evaluate trFNC estimation methods, at least regarding one specific aspect. First, we argue that trFNC estimation should include time-averaged connectivity (connectivity estimated using the whole temporal range of the time series) information, essentially the information at zero frequency of trFNC. Rather than treating trFNC as a contrast to time-averaged FNC, we consider it to be its generalization.

Averaging trFNC gives us its zero frequency (i.e., the time-averaged FNC). Thus, it is desirable for the average of the estimated trFNC to be close to the estimated time-averaged connectivity (i.e., connectivity calculated using the whole length of the data). So, the difference between averaged trFNC and time-averaged FNC (using Pearson correlation) can be used as an objective metric to evaluate different methods. So, we first estimate trFNC, using both SSB+SWPC and classic SWPC methods, and then average these matrices across the temporal domain. Then, we can calculate the mean-square distance between these averaged trFNC and time-averaged FNC (i.e., Pearson correlation using the whole temporal range of the data). The lower this value, the better the performance, as this would mean that the estimated trFNC is closer to the true trFNC at zero frequency. Note that this metric only compares the two methods regarding how well they can capture the zero frequency (i.e., constant) value of the true trFNC and does not provide any information about how well they can capture the temporal variation of trFNC.

### Real fMRI Data Analysis

We used both SSB+SWPC and SWPC to analyze a resting-state fMRI dataset. The complete information regarding the dataset used here can be found in previously published works (Faria et al., 2021; Kamath et al., 2018, 2019). The John Hopkins School of Medicine Institutional Review Board (IRB) approved the dataset gathering. All participants or their guardians signed a written informed consent. TC and FEP individuals were recruited through the John Hopkins Schizophrenia Center. For an individual to be considered part of the FEP group, they should have experienced their first episode of psychosis within 2 years before their enrollment. For this specific study, 183 subjects were included, with 94 belonging to the TC group. The 89 individuals belonging to the FEP group included individuals diagnosed with schizophrenia (*n* = 47), schizoaffective disorder (*n* = 10), schizophreniform disorder (*n* = 3), bipolar disorder with psychotic features (*n* = 21), major depressive disorder with psychotic features (*n* = 5), and not otherwise specified psychotic disorder (*n* = 3).

The fMRI data were prepared for analysis using the statistical parametric mapping (SPM12) toolbox within MATLAB 2019, which included the removal of the first five scans (to ensure signal equilibrium and participants’ adaptation to the scanner). Rigid body motion correction was performed using SPM to correct for subject head motion, followed by slice-timing correction to account for differences in slice acquisition timing. The fMRI data were then transformed into the standard Montreal Neurological Institute space using an echo-planar imaging (EPI) template and slightly resampled to isotropic voxels of size 3 × 3 × 3 mm^3^. Gaussian kernel smoothing with a full width at half maximum of 6 mm was applied to the resampled fMRI images. The smoothed datasets were used for the next steps. To decompose the data into a set of spatially maximal independent components, we used an approach based on independent component analysis (ICA) at its core (Calhoun et al., 2009). For this method, a brain mask was first obtained for each subject by finding all voxels with higher values (at all time points) than the total average of that subject’s BOLD data. Next, the group brain mask was calculated by finding the voxels that survived in all subjects’ masks. We used the group mask to extract the voxels in the brain for all subjects, normalized the brain data by calculating the z-score, and then applied principal component analysis (PCA) to reduce the temporal dimension of each subject brain data into 120. After concatenating all subjects’ reduced data across their reduced dimension, we applied a second PCA to reduce the dimension of the group data across the concatenated dimension to 100. Finally, we applied an ICA to the PCA results. We used the infomax algorithm for ICA (Bell & Sejnowski, 1995; Lee et al., 1999). To address the issue of the stability of ICA, we used the Icasso toolbox and selected the most stable run across the 20 ICA runs (Du et al., 2014; Himberg & Hyvarinen, 2003).

Next, we selected a subset of these 100 components as components of interest. We inspected each component visually and selected components that met the criteria for intrinsic brain networks based on the literature (Allen et al., 2014; Damaraju et al., 2014). we also consider the spectrum of these components’ time series when selecting components (Allen et al., 2011). This resulted in 52 components grouped into seven functional domains. Before estimating trFNC, we applied a bandpass filter to the data to limit the spectrum to 0.01 and 0.15 Hz. We used the Butterworth filter with optimal filter order for this step. We then use both SSB+SWPC and SWPC to estimate trFNC between all time series pairs. This results in a matrix of size 52 × 52 for each subject, each window index (i.e., *T* − 2Δ where *T* is the total number of time points and the window size is 2Δ + 1). First, we explored the spectral properties of the estimated trFNC using both SSB+SWPC and SWPC methods. Here, we explored the patterns of power spectral density (PSD) of the estimated trFNC. One pattern that has been reported many times for the PSD of brain signals is 1/*f*^*β*^ (*f* is frequency, and *β* is often called power law exponent). This pattern is closely related to a property called scale-freeness, which has been reported for many types of signals recorded from the human brain (He, 2011, 2014; Lee et al., 2021; Van de Ville et al., 2010). One way we can investigate this property is by first estimating the PSD. We used the function *pspectrum* implemented in MATLAB (The MathWorks Inc. version R2022b) to estimate PSD. We selected 0.5 for leakage (which controls the Kaiser window shape) and 0.01 for frequency resolution bandwidth. After estimating PSD for each subject and connectivity PSD, we fitted a power law function to the PSD using the *fit* function of MATLAB with ‘power1’ as the model. This resulted in one power law exponent for each subject and connectivity PSD. We compared the power law exponents estimated from SSB+SWPC and SWPC using a two-sample *t* test. The *p* values were corrected for multiple comparisons by controlling for the false discovery rate (FDR) using a linear step-up procedure (Benjamini & Hochberg, 1995).

To investigate the connectivity pattern shared between different subjects at different times, we used a clustering approach. It is important to note that as the connectivity matrices are symmetric, we only have 1,326 unique features (i.e., 52 × 51/2) for each matrix. We concatenated the estimated trFNC across all 183 subjects and windows, resulting in a big matrix with 183 × (*T* − 2Δ) rows and 1,326 columns. To summarize these trFNC matrices, we performed clustering using the k-means algorithm (Lloyd, 1982). As the data dimension is high, we used city block distance (also called Manhattan distance), as suggested by previous works for high-dimensional data (Aggarwal et al., 2001). We used the *k*-means++ algorithm for cluster center initialization (Arthur & Vassilvitskii, 2007). For each cluster number, the clustering was run 20 times with new initial cluster centroids, and the best clustering was chosen based on the run with the lowest within-cluster sum of distances between the points and centroids. To find the best cluster number, we use the elbow criterion on the within-cluster sum of distance. One metric we can calculate based on the clustering results is called dwell time, which simply shows how long each subject stays in a given cluster when it goes into that cluster. We have one dwell time value for each subject and each cluster. We compared this metric between TC and FEP groups while controlling for age, gender, and mean framewise displacement. We used robust fitting using the fitlm function implemented in MATLAB with ‘bisquare’ as the weight function, which has been shown to increase the sensitivity of the neuroimaging analysis (Wager et al., 2005). We corrected for multiple comparisons by correcting for FDR (Benjamini & Hochberg, 1995).

## RESULTS

### Simulation Results

**Figure 3.**
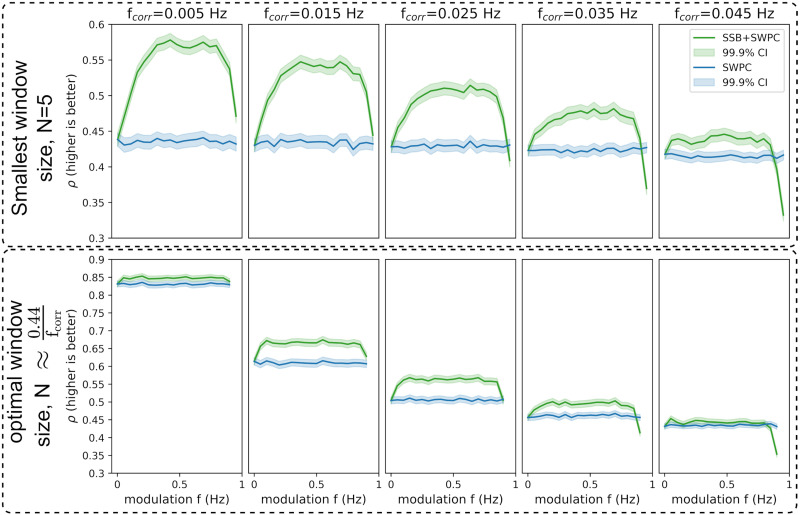
The results for the first set of simulations where the maximum value for true correlation is 0.7. Here, we show the correlation between the true connectivity values and the estimated values for both SWPC (blue lines) and SSB+SWPC (green lines). The solid lines are the average correlation across all realizations, while the shaded area is the 99.9% confidence interval (CI) of these averages. The first row shows the results of when we chose a very small window size while the second row shows the optimal window size results based on the true connectivity frequency (unknown in real-world settings). Each column shows the result from a specific connectivity frequency (i.e., *f*_*corr*_). Across both scenarios and almost all modulation frequencies, SSB+SWPC outperforms SWPC. The improvement can be as high as *ρ* ∼ 0.15 rho, which is a substantial improvement.

**Figure 4.**
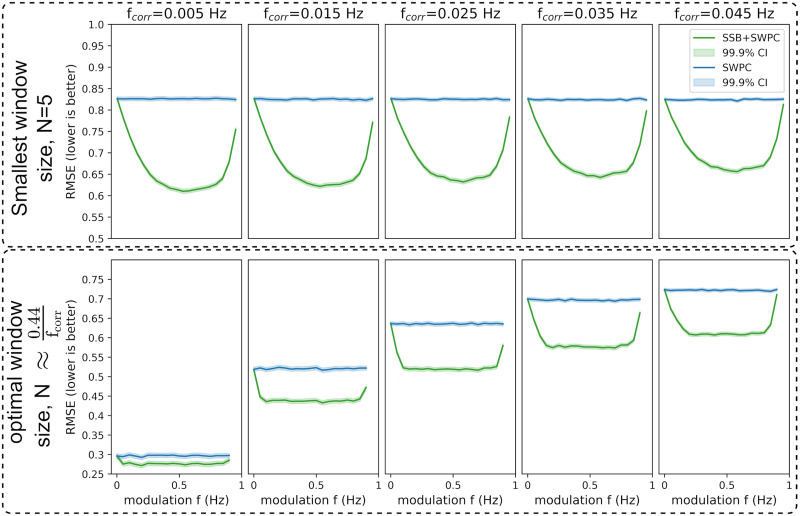
The results for the first set of simulations where the maximum value for true correlation is 0.7. Here, we are showing the root-mean-square error (RMSE) between the true connectivity values and the estimated values for both SWPC (blue lines) and SSB+SWPC (green lines). Lower RMSE means that the estimand is closer to the true value; therefore, the estimation is better. The solid lines are the average correlation across all realizations, while the shaded area is the 99.9% confidence interval (CI) of these averages. The first row shows the results of when we chose a very small window size while the second row shows the optimal window size results based on the true connectivity frequency (unknown in real-world settings). Each column shows the result from a specific connectivity frequency (i.e., *f*_*corr*_). Across both scenarios and almost all modulation frequencies, SSB+SWPC outperforms SWPC. The improvement can be as high as almost 0.2 points, which is a substantial improvement.

Two sets of simulations were designed to evaluate the proposed methods in two different scenarios. For the first scenario, we generated two random time series to have a time-resolved correlation that changes as a sinusoid with frequency *f*_*corr*_. Using different analysis parameters, we then used the proposed method, SSB+SWPC, and classical SWPC to estimate the time-resolved correlation between the time series. We can compare the two methods by comparing the estimated correlation time series with the true time series. We used both Pearson correlation and RMSE as the performance metrics here. Figure 3 and Figure 4 show the results for different simulation parameters. Each row shows the results from one specific approach to window selection. The first row shows the results for when the window size is fixed (equal to 5 time points, which is quite small), while the second row shows the results for the case where the window size is selected optimally based on the connectivity frequency or *f*_*corr*_ (which is unknow for real data). Each column shows the result for a specific *f*_*corr*_, while the *x*-axis of each figure is the modulation frequency (i.e., *f*_*m*_ a free parameter of SSB+SWPC). For this specific simulation scenario, the two signals’ spectrum had frequency content in the range [0, 0.1] Hz.

As we can see here, SSB+SWPC (green line) outperforms SWPC for most frequency modulation values based on both metrics. The performance improvement of using SSB+SWPC is more noticeable if we use a very small window size (five samples here), but it exists even for optimal window size. This observation is quite logical as using a small window size, the high-pass filter of SWPC removes a lot of information from the activity signals in the classical SWPC, while in SSB+SWPC, the low-frequency information is modulated to higher frequencies. Suppose we select the optimal window size (by selecting the window size so that the low-pass filter cutoff is equal to the connectivity frequency). In that case, we see that the improvement is less pronounced, but note that if we do not know the frequency of connectivity, as would be the case in a practical setting since it is the quantity to estimate, we cannot know the optimal window size. Therefore, one can argue that it is quite beneficial to use window small window sizes to be able to estimate connectivities across broader frequency ranges. Another observation we can make based on these two figures is that most modulation frequency values result in a better estimation performance, which means that the impact of the choice of this parameter is somewhat lessened. We should keep in mind to select this parameter in a way that aliasing (modulation of a signal past half of the sampling frequency, i.e., *F*_*s*_/2) does not happen. This aliasing is likely why the performance of SSB+SWPC is worse than SWPC for that right-most column in Figure 3 and Figure 4 for high modulation frequency values. To summarize, we just make sure that the modulation frequency is less than *F*_*s*_/2 − *b* where *b* is the highest frequency of the activity signals. In resting-state fMRI studies, we often pass the activity signals through a bandpass filter. Therefore, this *b* is determined by the designed bandpass filter.

Here, for the completion’s sake, we have included the results of RMSE (Figure 4), as it is used more often when evaluating estimators. The results of this metric align with the results of using Pearson correlation as the evaluation metric (Figure 3) and show that SSB+SWPC outperforms classic SWPC across different window sizes and connectivity frequencies. But we want to point out that for the specific application of interest in this paper (i.e., whole-brain temporal network construction), we care more about preserving the connectivity patterns between different nodes than the actual values of the connectivity themselves. This reasoning is also why clustering approaches for summarizing the time-resolved functional connectivities make sense. These pattern recognition approaches aim to capture the overall pattern of the connections and group them into several clusters instead of capturing the absolute values.

**Figure 5.**
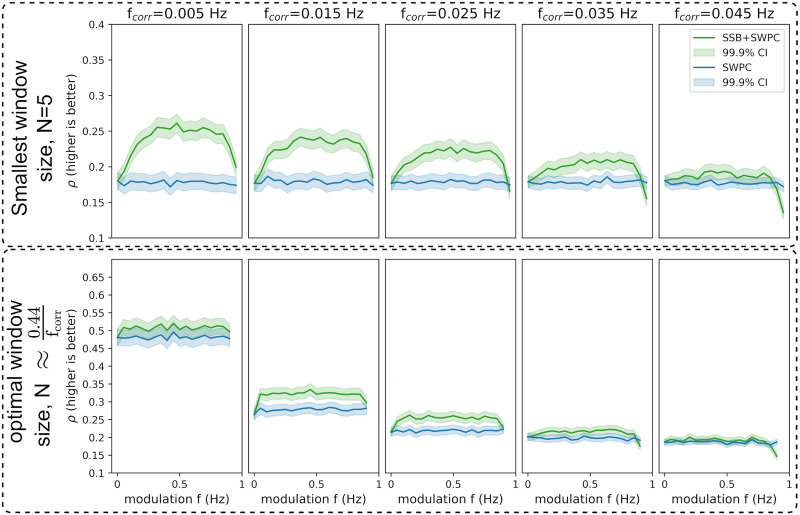
The results for the first scenario of simulations where the maximum value for true correlation is 0.3. Here, we show the correlation between the true connectivity values and the estimated values for both SWPC (blue lines) and SSB+SWPC (green lines). The solid lines are the average correlation across all realizations, while the shaded area is the 99.9% confidence interval (CI) of these averages. The first row shows the results of when we chose a very small window size while the second row shows the optimal window size results based on the true connectivity frequency (unknown in real-world settings). Each column shows the result from a specific connectivity frequency (i.e., *f*_*corr*_). The point of this simulation was to show that even when the maximum amplitude of correlation is low (compared to Figure 3), SSB+SWPC outperforms classic SWPC across different parameters.

We also repeated this scenario for the case where the maximum amplitude of the connectivity is not as high as in Figure 3 and Figure 4 (i.e., 0.7). Figure 5 shows the case where the maximum amplitude of the connectivity is 0.3. Similar to the previous cases, we can see here that SSB+SWPC outperforms classic SWPC across different simulation parameters. It is important to note that the decrease in the correlation of both SSB+SWCP and SWPC methods in Figure 5 (when compared to Figure 3) is expected, as by lowering the maximum amplitude of the connectivity time series, we are reducing the overall power of connectivity. But even with this decrease in performance across the board, SSB+SWPC still outperforms SWPC.

**Figure 6.**
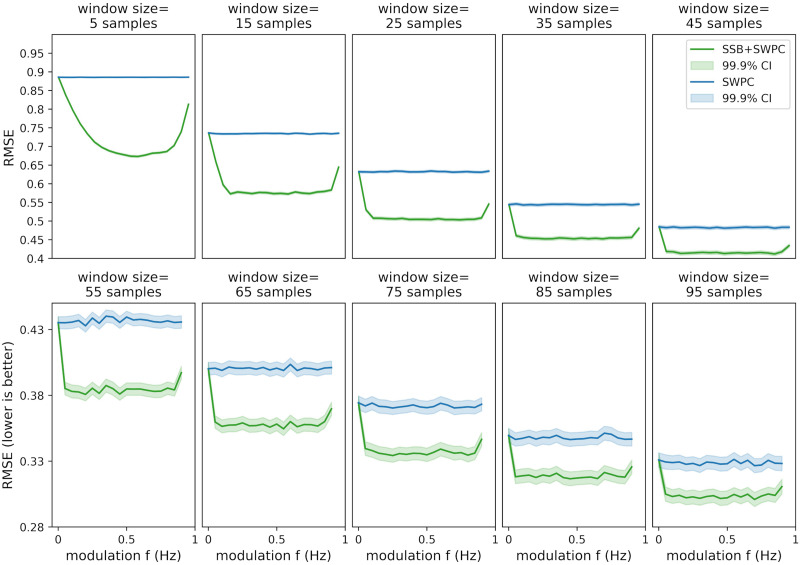
The results for the first scenario of simulations where true correlation does not change with time and is always zero. Here, we are showing the root-mean-square error (RMSE) between the true connectivity values and the estimated values for both SWPC (blue lines) and SSB+SWPC (green lines). The solid lines are the average correlation across all realizations, while the shaded area is the 99.9% confidence interval (CI) of these averages. Each figure shows the results of a specific window size. As expected, larger window sizes show better results. This result is expected because the true correlation does not change with time. Even in this case, we can see that across the board, SSB+SWPC shows improvement in the results for all modulation frequencies.

One final case for the first scenario is when the connectivity frequency and amplitude are zero. In other words, if the connectivity does not change with time and is zero for all time points. Figure 6 depicts the results of this simulation. As we can see, even when the assumption of time-varying correlation does not hold, and correlation is equal to zero across the whole temporal range of the data, SSB+SWPC outperforms classic SWPC using different window sizes. This improvement can be explained by the fact that in SSB+SWPC, the frequency contents of the pairs of the signals can be modulated out of the stop band frequencies of the high-pass filter of SWPC, that is, the correlation estimator (SWPC) has more data available to work with, which in turn translates into better performance. This phenomenon happens regardless of the frequency and amplitude of the true correlation as long as the signal is band-limited.

**Figure 7.**
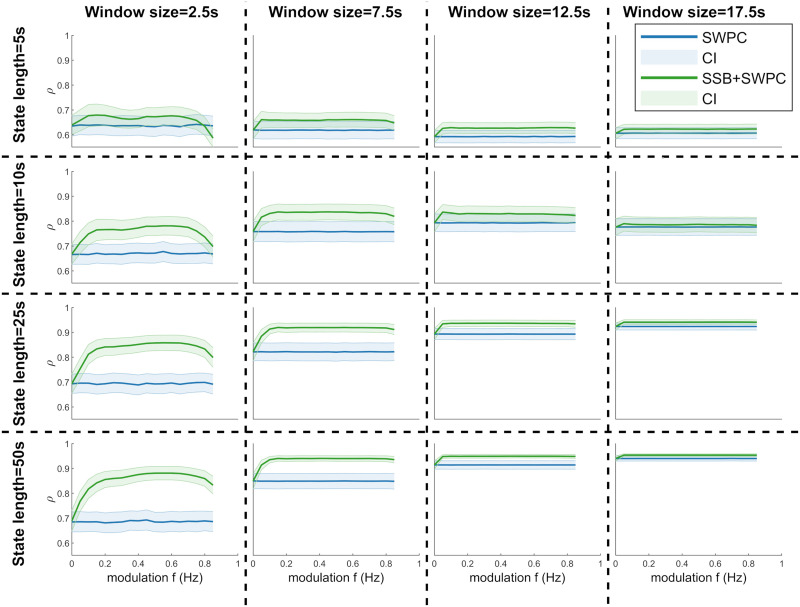
The results of the second set of simulations. Here, we compare how well we can estimate FNC states using SSB+SPWC (green lines) and SWPC (blue lines) methods. Each row shows the result for one specific state length, while each column shows the result when using one specific window size. We can see that SSB+SWPC outperforms SWPC again across almost all parameters. The performance improvement is more noticeable for smaller window sizes. This observation makes sense as the larger the window size, the lower the cutoff frequency of the high-pass filter of SWPC, meaning we are filtering out less information. Therefore, SSB+SWPC and SWPC results are more similar for larger window sizes.

For the second simulation scenario, we aimed to evaluate the performance of a pipeline that breaks the data into temporal connectivity states by clustering trFNC using both SSB+SWPC and SWPC methods. Here, we examined how well we can estimate the final connectivity states rather than the trFNC values themselves (examined in the previous simulation). This simulation is more relevant as we break the data into temporal connectivity states in the actual analysis of the real resting-state fMRI data, and most of the metrics are calculated for those brain states. Each subfigure in Figure 7 shows the final results for one simulation. The rows and columns show the results from different state lengths and window sizes, respectively.

It can be seen in Figure 7 that using SSB+SWPC improves the performance of the pipeline compared to SWPC across almost all of the different simulation parameters. Similar to the previous simulation, this improvement is more substantial for the smallest window size. For the case of the smallest window size and state length, SSB+SWPC performs worse than SWPC for larger modulation values, but this is likely caused by the aliasing issue we discussed above and can be avoided by choosing an appropriate modulation frequency value. By combining the results of the first simulation scenario (Figure 3 to Figure 6) with the second simulation (Figure 7), we can see that the performance is higher across the board for both methods in the second simulation. The reason is that we are estimating the trFNC values in the first simulation. In contrast, for the second simulation, the trFNC values are clustered into a limited number of clusters (two here). Therefore, we are averaging a lot of data to get the states, meaning that some of the noise and error of trFNC estimation are averaged out. Both simulation results showed that using SSB+SWPC, we can achieve better performance compared to using classic SWPC in analyses that are based on estimating trFNC.

### Real fMRI Data Analysis

**Figure 8.**
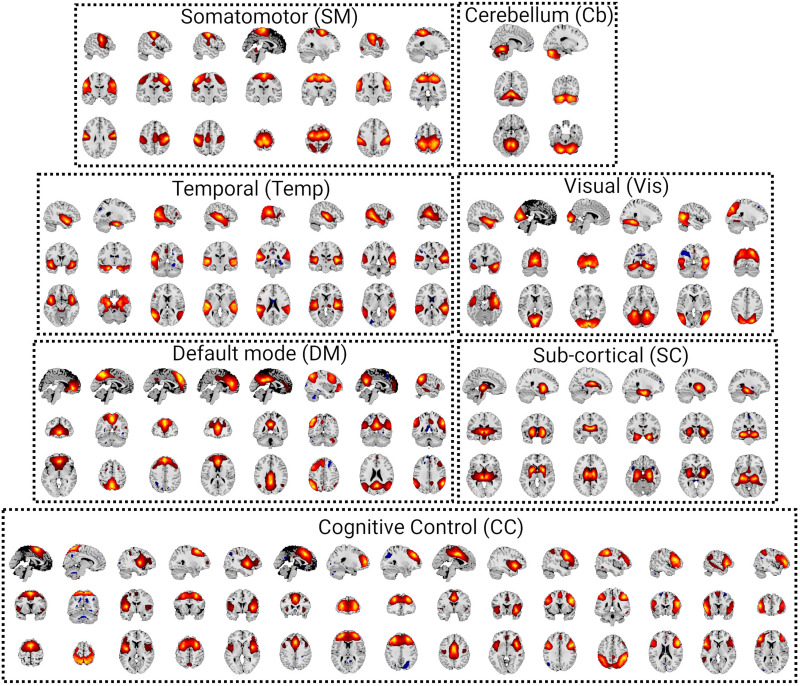
Spatial map of all 52 components grouped into seven functional domains: Somatomotor (SM), Cerebellum (Cb), Temporal (Temp), Visual (Vis), Default mode (DM), Subcortical (SC), and Cognitive control (CC) networks.

After standard preprocessing, we decomposed the dataset into 100 maximally spatially independent components using the GIFT toolbox and visually selected 52 components based on their spatial maps and spectral properties. These 52 components were then grouped into seven functional domains: Visual (Vis), Somatomotor (SM), Temporal (Temp), Cerebellum (Cb), Default mode (DM), Cognitive control (CC), and Subcortical (SC) networks. Figure 8 shows the spatial maps of these 52 components and their grouping. We then used a window size of seven samples (equal to 14 seconds as TR is equal to 2 seconds) to estimate trFNC using both SSB+SWPC and SWPC methods. The reason we used this short window size was to emphasize the benefit of using SSB+SWPC, as using a small window size, important low-frequency information will be filtered out in SWPC but not in SSB+SWPC. To get the optimal modulation value for SSB+SWPC, we first calculate the cutoff frequency of the high-pass filter of SWPC. As we are using a rectangular window here, the approximate −3 dB cutoff frequency of this high-pass filter can be calculated using this formula:

$$F_{\text{cutoff}}=\frac{0.88}{\sqrt{N^{2}-1}}F_{s}$$ (6)

*F*_*s*_ is the sampling frequency (i.e., 0.5 Hz), while *N* is the window size (i.e., 7). For information about how we have arrived at this formula, refer to the Supporting Information. Using this formula, we get 0.0635 Hz as the cutoff frequency for the high-pass filter of SWPC. As the lowest frequency of the activity signals is 0.01 Hz (the lower bound of the bandpass filter applied to the data), we have to modulate the activity signals by 0.0625 Hz (i.e., 0.0635–0.01) to make sure the high-pass filter of SWPC does not filter important low-frequency information of the activity signals. Next, to summarize the trFNC time series, we applied *k*-means to cluster the results. To select the cluster number, we used the elbow criterion method. First, we ran the clustering for cluster numbers from 1 to 30, recorded the sum of square error between cluster centroids and the data in each cluster, and averaged these values across cluster numbers. This step resulted in one value for each clustering number. Next, we use the elbow criterion to select the best cluster number. The elbow criterion is usually qualitative, but we used a more quantitative method here. In short, we fitted two lines to the average within-cluster distance curve. One line fitted to the values belonging to small cluster numbers and one line fitted to the larger cluster numbers. Then, the intersection of these two lines can be viewed as the elbow of the curve and be selected as the best cluster number. The intersection of the two lines determines that 4 is the best cluster number based on the elbow criterion (see Figure S2 in the Supporting Information).

### Method Evaluation Using fMRI Data

**Figure 9.**
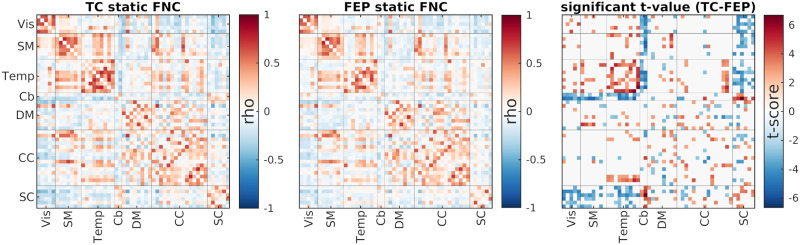
sFNC for both TC (left figure) and FEP (FEP; middle figure). The right figure shows the significant differences between the two groups of sFNC evaluated using a two-sample *t* test (corrected for multiple comparisons using the FDR correction approach). TC individuals have stronger anticorrelated (i.e., more negative) connections between sensory areas (Vis, SM, and Temp) with both Cerebellum and Subcortical regions.

As mentioned in the Methods section, we can evaluate a method for estimating trFNC by comparing how well it captures the time-averaged FNC. We can do this by comparing the average trFNC with FNC calculated using the whole temporal range of data using the Pearson correlation estimator (the term static FNC is often used to describe this metric). Figure 9 shows the static FNC (sFNC; FNC estimated using the whole temporal range of the data) for both TC and FEP individuals in addition to the significant (*p* value < 0.05) difference between the two groups. We can see here that TC individuals have stronger anticorrelated connections between the sensory functional domains (i.e., visual, sensorimotor, and temporal domains) and cerebellar and subcortical domains. Additionally, we can see that the TC group has a stronger connection within the temporal functional domain.

**Figure 10.**
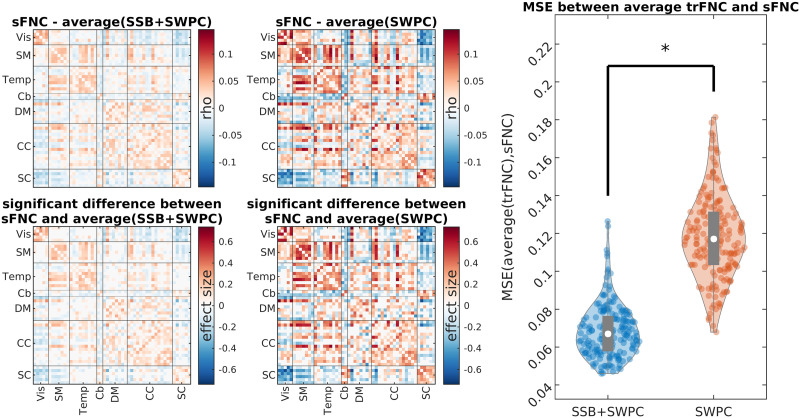
Evaluation of the estimation methods based on how well they can capture time-averaged information. The two figures in the top row (left half figure) show the difference between averaged trFNC estimation and static (time-averaged) correlation for both SSB+SWPC and SSB methods, respectively. The bottom row (left half figure) shows the Cohen’s *d* effect size for significant differences between averaged trFNC and static correlation. The statistical test used here was a *t* test with an alpha value equal to 0.01. We corrected for multiple comparisons using an approach that corrects for FDR. The right half of the figure shows the mean squared error between averaged trFNC and time-averaged correlation for the two methods (MSE calculated across all component pairs for each subject). We used the two-sample *t* test to compare the results between the two estimators. As we can see here, the average of SSB+SWPC is significantly (*p* < 0.01) closer to sFNC than the average of SWPC, that is, SSB+SWPC can capture the zero frequency content of connectivity better than SWPC.

The closer the average trFNC and time-averaged FNC are, the better the estimation of trFNC is, at least regarding the content of trFNC at zero frequency. The reason behind this argument is that trFNC is not the opposite of time-averaged FNC (i.e., sFNC) but rather a generalization of it. Time-averaged FNC is essentially an estimate of connectivity at the frequency of zero. At the same time, trFNC estimates the connectivity across a broader spectrum, including zero frequency (at least in the case of SWPC). Therefore, it makes sense for us to expect that the average of trFNC estimation using both methods should be close to time-averaged FNC that is estimated directly using Pearson correlation over the whole temporal range of the data. Figure 10 shows the difference between averaged trFNC and time-averaged FNC for both SSB+SWPC and SWPC methods. As we can see, the mean squared error between average trFNC and sFNC is significantly lower for the SSB+SWPC method than the SWPC method (right-most figure), showing that SSB+SWPC outperforms SWPC. This result demonstrates that SSB+SWPC can capture the zero frequency content of trFNC better than SWPC. However, this does not tell us how well it can estimate trFNC of frequencies above zero. It is quite challenging to evaluate the performance of any estimator for those frequencies without having access to the true values.

**Figure 11.**
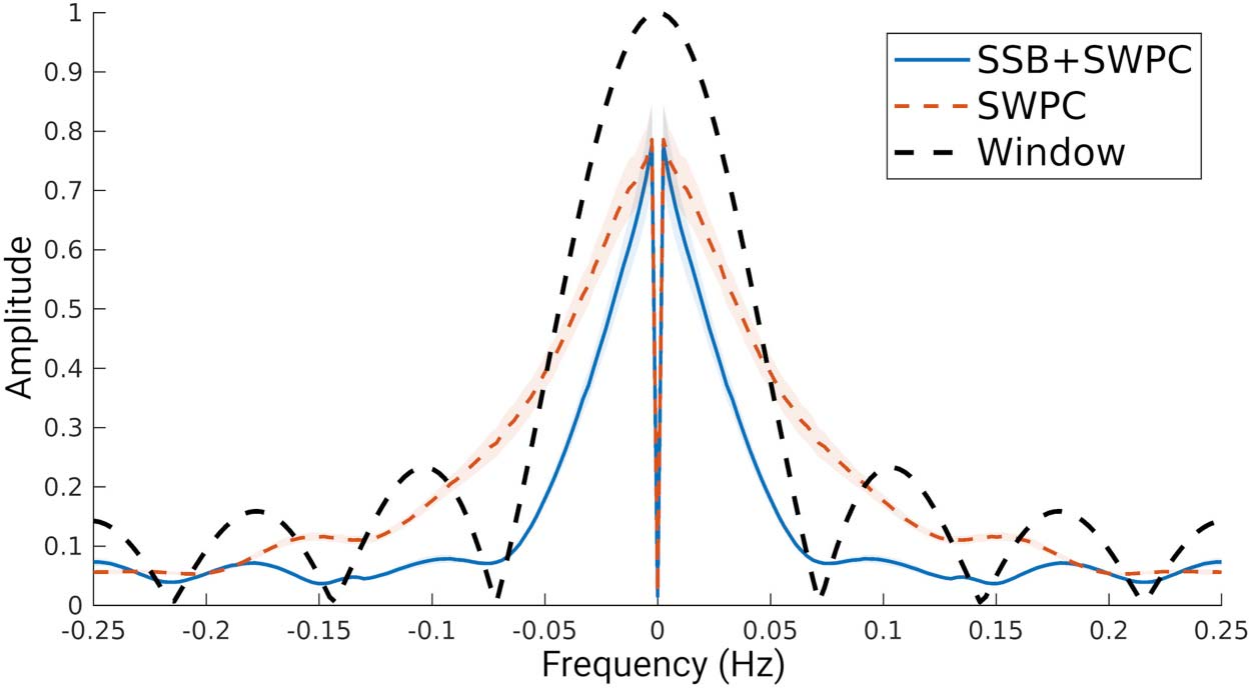
The average spectrum of trFNC estimated using both SSB+SWPC and SWPC methods. As we can see here, the spectrum of the SSB+SWPC estimate follows the transfer function of the sliding window better than estimates of SWPC. Note that as the sliding window is the last subsystem of both methods, the output (i.e., trFNC estimation) should be somewhat similar to the transfer function of the window. This is more important in the side lobes. We can see here that the SWPC spectrum has high values for at least the first side lobe.

Another metric we can look at for qualitative evaluation of trFNC estimation methods is the spectrum of the estimated trFNC time series. As both methods being compared here, namely, SSB+SWPC and SWPC, have a low-pass filter as their last subsystem (i.e., the sliding window part), we can compare the average spectrum with the response function of this low-pass filter. Ideally, we want a spectrum that is quite similar to the response function of the filter. Figure 11 shows the average spectrum across all subjects and connectivity pairs. As we can see here, the SSB+SWPC average spectrum is more similar to the response function of the low-pass filter compared to that of SWPC. This is likely because we have selected a very small window size (7 time points), which results in the removal of important low-pass information of the activity signals, which in turn means the signal-to-noise ratio is lowered. As in the SSB+SWPC, the activity signal is modulated to higher frequencies and the low-frequency information is not removed by the high-pass filter portion of SWPC.

**Figure 12.**
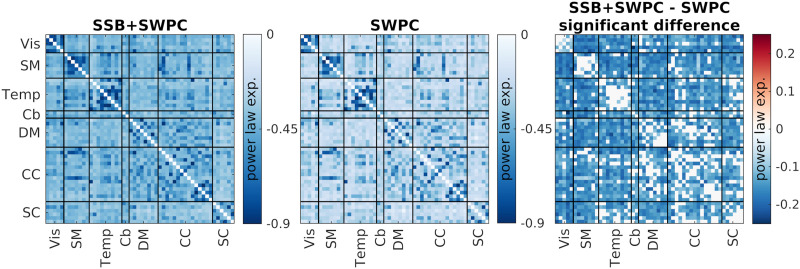
Estimated power law exponent for both SSB+SWPC and SWPC methods and their differences. The left figure shows the average power law exponent values (across subjects) for trFNC estimated using SSB+SWPC, while the middle figure shows the same measure for SWPC. The right figure shows the significant difference between the power law components estimated using both methods. We used the two-sample *t* test here to find the significant difference between the results of the two methods (*p* < 0.01) and corrected for multiple comparisons using a method that corrects for FDR. A power law exponent closer to −1 might point to a signal with higher complexity, while an exponent closer to zero (i.e., a flatter power spectrum) means that the signal is closer to white noise, at least regarding the power at different frequencies.

The scale-free property of the trFNC time series is investigated by fitting a power law function (*y* ∼ *x*^*β*^) to trFNC power spectral density. *β* is called the power law exponent, and the closer it is to −1, the more complex and scale-free the signal is (Lee et al., 2021). Furthermore, according to the Wiener-Khinchin theorem, the power spectrum can be understood as the Fourier transform of the autocorrelation function for a wide-sense stationary random process (Wiener, 1930). If the power spectrum exhibits a steeper slope (indicated by a more negative β value), it indicates a slower and weaker autocorrelation, which would, in turn, mean reduced redundancy. On the other hand, If *β* is closer to 0, the signal has similar spectral properties to white noise (noise with similar power at all frequencies). Figure 12 shows the average power law exponent for the trFNC time series estimated using both SSB+SWPC and SWPC estimators (the left and middle figures). The right figure shows the difference between the two estimators’ power law exponent values. We can make two observations here; first, we see that intradomain connectivity (especially for Vis, SM, and Temp functional domains) has power exponent values closer to −1 than interdomain trFNCs based on both SSB+SWPC and SWPC results. This result might highlight the higher complexity of these specific connectivity time series. The second observation we can make is that trFNC estimated from SSB+SWPC shows a significantly lower power law exponent (i.e., closer to −1) than that of SWPC across many interdomain connections. This might be related to our hypothesis (supported by our simulations) that SSB+SWPC has a higher signal-to-noise ratio (noise here being white noise that has a flat spectrum) as it does not filter out important low-frequency activity information when estimating trFNC.

### Real fMRI Data Time-Resolved Results

**Figure 13.**
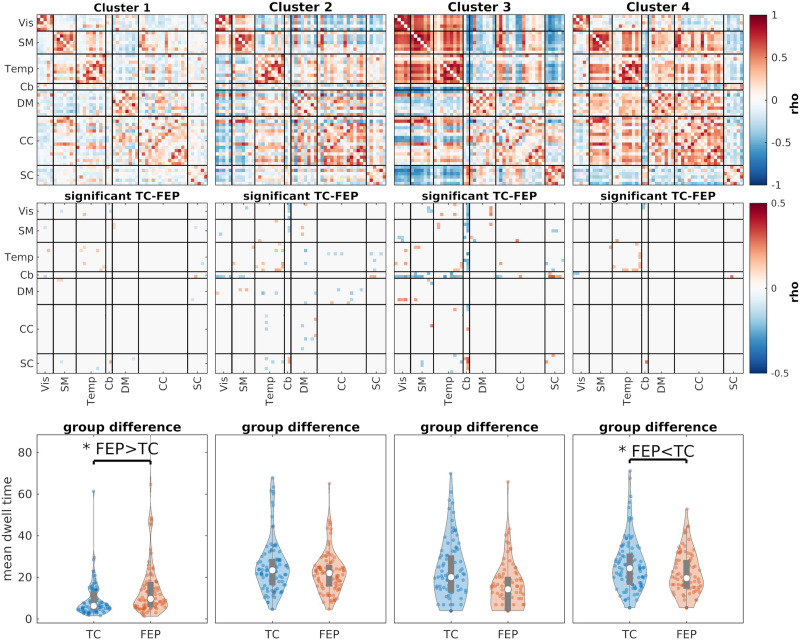
The clustering results for SSB+SWPC. The first row shows the four cluster centroids. The second row shows the cells that are significantly different between TC and FEP (subject specific) cluster centroids. We used the two-sample *t* test here. The last row shows the comparison of the mean dwell time for each cluster between the two groups. For these results, we used linear regression to find significant differences between the two groups, including age, gender, and mean framewise displacement as nuisance regressors. Clusters 1 and 4 show significant differences between FEP and TC (*p* < 0.05) mean dwell time. FEP individuals generally stay more in state 1, while TC individuals significantly stay more in cluster 4. Cluster 1 shows a more disconnected (i.e., less modular) connectivity pattern (compared to state 4). This observation is in line with the previous studies that have reported disconnection in individuals with schizophrenia. In addition, TC individuals stay more in state 4, which is more modular than state 1. State 4 also shows negative interdomain connectivity between SC (and Cb) and all other functional domains.

**Figure 14.**
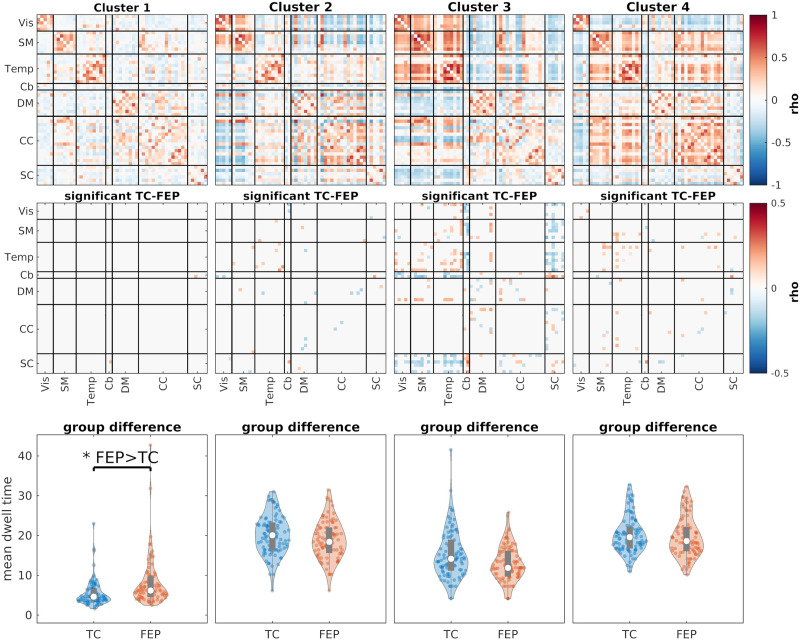
The clustering results for SWPC. The first row shows the four cluster centroids. The second row shows the cells that are significantly different between TC and FEP (subject specific) cluster centroids. We used the two-sample *t* test here. The last row shows the comparison of the mean dwell time for each cluster between the two groups. For these results, we used linear regression to find significant differences between the two groups, including age, gender, and mean framewise displacement as nuisance regressors. The last row shows the comparison of the mean dwell time for each cluster. Similar to SSB+SWPC results (Figure 13), FEP individuals stay more in state 1, which has a weaker connection between different parts of the brain compared to other states. But unlike SSB+SWPC results, cluster 4 dwell time is not significantly different between the two groups.

We can also use the clustering results to compare the two groups (i.e., TC and FEP). One metric we can use for this comparison is the mean dwell time. In short, this value determines how many time points, on average, each subject stays in one cluster once it goes in that cluster. Mean dwell time can be used to measure how much each subject’s data sticks to a given FNC state. Figure 13 shows the clustering results for SSB+SWPC while Figure 14 shows the results using SWPC. Based on these two figures, we see that two clusters show significant differences between the two groups’ mean dwell time in SSB+SWPC results.

In contrast, only one cluster shows a significant difference between the two groups for SWPC results. Note that this difference between the two methods’ results should not be considered as us saying that SSB+SWPC is outperforming SWPC. In addition, cluster 4 shows a stronger positive connection between SM/Temp domains and DM/CC domains. And SC components show negative connectivity with all other functional domains other than Cb. These differences are more pronounced in SSB+SWPC results (Figure 13) than in SWPC results (Figure 14).

## DISCUSSION

In this article, we proposed a method to overcome one specific shortcoming of the sliding window Pearson correlation estimator, namely, the fact that if a small window size is chosen, some important low-frequency content of the activity signal might be filtered out, resulting in a subpar estimation performance. In other words, using shorter window sizes is equivalent to a lower signal-to-noise ratio, as we remove good signals using the high-pass filter of SWPC (Hindriks et al., 2016; Leonardi & Van De Ville, 2015). This is possibly the biggest shortcoming of SWPC, at least for rsfMRI analysis, because of two points: firstly, as we do not know the frequency content of the functional connectivity (most of the research has been on activity spectrum and not the connectivity spectrum), we want to select a small window size so that we can capture the connectivity across a broad range of frequencies. Secondly, much of the important information of rsfMRI activity resides in low frequencies (Biswal et al., 1995; Kalcher et al., 2014; Van Dijk et al., 2010). Although recent studies have increasingly emphasized the presence of rich information in higher frequencies within rsfMRI, challenging our prior understanding (Chen & Glover, 2015; DeRamus et al., 2021; Kajimura et al., 2023), the low frequencies contribute the greatest power it seems (Ciuciu et al., 2014). Based on these two points, the method proposed here that allows us to select smaller window sizes for SWPC while improving its estimation can be quite useful. We demonstrated the superior performance of the SSB+SWPC (i.e., the proposed method) compared to the traditional SWPC approach and showcased its application for analyzing an rsfMRI dataset.

For any method aimed at enhancing parameter estimation, it is highly desirable to comprehend why the method outperforms other approaches based on its mathematical formulation. In the Methods section, we explained why our methods should allow us to use smaller window sizes for SWPC if the data is band-limited. In short, the coupling subsystem of SWPC is a multiplication in the time domain, which translates into a circular convolution in the frequency domain (Faghiri et al., 2022b). Therefore, as long as we modulate both input signals of SWPC (i.e., activity signals) with the same frequency value while keeping the spectrum symmetric around zero, the estimated connectivity will not change. Because of this, we can reduce the window size of SWPC without worrying that the high-pass filter of SWPC is filtering out important low-frequency information of the activity signals. This is purely based on the mathematical formulation of SSB+SWPC, and therefore, even without empirical analysis, we can argue that our method should, at least in theory, outperform SWPC. One assumption is that the signal should be band-limited, which is the case for rsfMRI data. Note that this improvement in the performance is based on the bias of the estimator and not its standard error. Deriving the formula for the standard error of SWPC is not an easy undertaking and is beyond the scope of this study.

In addition to the theoretical reason why the SSB+SWPC method outperforms SWPC, we also showed the improvement our proposed method offers by using two specific simulation scenarios. First, we showed that if our signals are band-limited, and the connectivity is a sinusoid signal with a specific frequency, SSB+SWPC can improve the estimation of time-resolved correlation across most parameters (correlation frequency and modulation frequency). The improvement is pronounced when we use a very small window size, up to *ρ* ∼ 0.15, which is quite high. But even if we use the optimal window size based on the correlation frequency (not known in real-world settings), SSB+SWPC improves the estimation. The reason behind this is that even if we use the largest window size possible that allows us to capture the connectivity of a specific frequency, this window size might still be smaller than the window size that does not filter out important low-frequency information of the activity signal.

Another way we can view this is that we have two (usually unknown) data parameters that we have to optimize for: the frequency of the activity signal and the frequency of the connectivity signal. However, the SWPC method technically has only one parameter, the window size, that we need to select to match the two parameters of the data. The window size should be large enough so that the high-pass filter subsystem of SWPC does not filter out low-frequency information of the activity signal. On the other hand, it should be short enough to capture the connectivity. Depending on the frequency profile of the activity and connectivity signals, these two goals might not be possible with only one parameter. SSB+SWPC, our proposed method, adds an additional analysis parameter in the form of the modulation frequency, allowing us to select the window size without worrying about the activity signal frequency profile. Based on the selected window size, we can modulate the activity signal so that the high-pass filter of SWPC does not filter any important information. In short, the SSB+SWPC has two analysis parameters we can tune to match the data parameter. Note that SWPC technically has another parameter, window shape (e.g., rectangular or Gaussian). But that parameter is minor as it impacts the SWPC filters’ transfer function shape (i.e., the transition between passbands and stopbands) rather than their respective cutoff frequencies most of the time. In addition, we can also have a window shape for SSB+SWPC.

The second simulation showcases how SSB+SWPC performs in a more complete pipeline for analyzing trFNC. Usually, the data we work with has many individual subject data included in it in addition to temporal and spatial information. Many trFNC analysis pipelines aim to first construct networks from these data and then capture a specific number of FNC patterns shared between different subjects at different temporal locations and scales. We often do this with the help of a clustering or dimensional reduction approach. In the second simulation, we assumed that the data occupies a limited number of correlation patterns at different times, often called FNC states in the field. Then, we evaluated the performance of a pipeline that uses SSB+SWPC for estimating time-resolved correlation and *k*-means for the clustering step and compared the performance with a similar pipeline that uses classic SWPC. We showed that across different parameters of data (correlation state length) and analysis parameters (window size and modulation frequency), SSB+SWPC outperforms SWPC. Similar to the previous simulation, the improvement provided by SSB+SWPC is greater when we use the smallest window size. One observation we made about the result of this simulation is that the performance values are higher than the first simulation for both SSB+SWPC and SWPC methods. The reason for this is that in the first simulation, we are estimating the time-resolved correlation itself, while in the second simulation, we are estimating the handful of shared correlation patterns (i.e., states), meaning we have much more data to estimate a smaller number of values. Therefore, the performance is better across the board. It is important to note that the second simulation is closer to the actual analysis that is often used, as we rarely care about the trFNC values across all time points, nodes, and subjects. We care more about the patterns repeated many times in the data as a whole.

**Figure 15.**
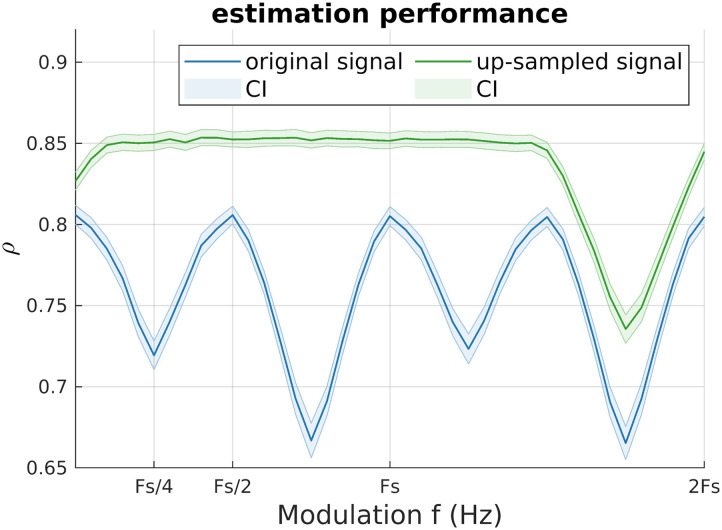
Impact of up-sampling on SSB+SWPC estimation performance when the signals have content at all frequencies. The *y*-axis shows the correlation between the estimated and the true value, meaning higher is better. The blue lines show the correlation for the original signal, while the green line shows the correlation for when up-sampling was used before SSB+SWPC. The shades show the 95% confidence intervals (CI), corrected for multiple comparisons. We can see that using up-sampling allows us to use the estimation improvement provided by SSB+SWPC.

As we mentioned, one assumption of SSB+SWPC is that the signal should be band-limited. So, does this mean SSB+SWPC cannot be used for signals with information at every frequency? Not necessarily. As we know, if we up-sample a time series that is sampled with the sampling frequency *F*_*s*_, by *n*, we will have a new signal with a spectrum in the range [−*nF*_*s*_/2, +*nF*_*s*_/2] but the information is band-limited to the spectral range [−*F*_*s*_/2, +*F*_*s*_/2]. Therefore, we can make any signal band-limited by using up-sampling, allowing us to use SSB+SWPC on even white signals. We leave the in-depth exploration of this statement for a future study and only do a simple simulation here. Assume we simulate a pair of time series by drawing 1,000 samples from a multivariate normal distribution with mean 0, variance 1, and covariance equal to 0.7 × cos(2*π* × 0.01 × *t*); that is, the correlation changes with time. The sampling frequency is 1 Hz. Note that the samples are independent and identically distributed (iid) random variables, so the generated signals have content at all frequencies. Next, we can use SSB+SWPC to estimate the time-resolved connectivity on the original and up-sampled data (up-sampling factor 4). We can evaluate the performance of the estimators by calculating the correlation between true and estimated values. Figure 15 shows the results of a simulation repeated 1,000 times for different modulation frequencies, with results of classic SWPC being included at modulation frequency zero. As we can see in Figure 15, SSB+SWPC improves the estimation if it is paired with up-sampling. This improvement is significant across all modulation frequencies, including zero frequency, interestingly. Based on this figure, we see that if we do not use the up-sampling, SSB+SWPC results in worse estimation compared to typical SWPC (results at zero frequency) because of the aliasing that happens at all modulation frequencies. The periodic nature of this result is possibly caused by the fact that the spectrum is cyclic for discrete signals. The dip that happens in the up-sampled results (i.e., green line) is because of aliasing, which was also apparent in Figure 3. To conclude, applying up-sampling on a signal with content at all frequencies enables us to use SSB+SWPC and improve the time-resolved correlation. We leave the more in-depth exploration of this idea to future works, especially because, based on Figure 15, up-sampling can improve classic SWPC results (look at zero modulation frequency).

We used a pipeline based on ICA to decompose the preprocessed rsfMRI data from all individuals into 100 spatially maximally independent components. We then selected 52 components and grouped them into seven functional domains (see Figure 8). trFNC was estimated using both SSB+SWPC and SWPC methods. We first investigated how close the average trFNC was to time-averaged FNC, calculated using the sample Pearson correlation estimator using the whole temporal range of each subject’s data. Figure 9 shows the static FNC for both TC and FEP groups and the significant difference between the groups. We see that the TC group shows more negative connectivity between Subcortical and sensory regions (VIS, SM, and Temp) compared to the FEP group.

Similarly, Cb regions have stronger negative FNC than sensory regions. This aligns with previous research (Damaraju et al., 2014). We also see that the TC group has stronger connectivity in the temporal domain compared to the FEP group. Crossley et al. reported dysconnectivity in the temporal lobe (Crossley et al., 2009). We also showed that the average trFNC is closer to sFNC if we use SSB+SWPC significantly in Figure 10, which means that sFNC is captured more accurately in trFNC if we use SSB+SWPC. This is an important point, as an ideal trFNC estimation should include an accurate estimation of sFNC, that is, trFNC is not an opposing view of sFNC but rather should complement it.

We also explored the spectral properties of the estimated trFNC. Figure 11 depicts the average fast Fourier transform of estimated trFNC using both SSB+SWPC and SWPC methods and the transfer function of the low-pass filter of SWPC. Note that as the last subsystem of SWPC is this low-pass filter, we expect the output (i.e., trFNC) to have a similar spectrum to the transfer function of the low-pass filter. Figure 11 shows that trFNC estimated using SSB+SWPC is more similar to the transfer function than trFNC estimated using classic SWPC. The difference is most apparent in the side lobes of the transfer function, possibly pointing to the fact that SWPC estimates are more noisy. The power spectra of neural activity can provide much meaningful information regarding the human brain (Gao, 2016), but not many studies have explored the spectral properties of the connectivity time series. In this work, we investigated the similarity of the trFNC power spectrum to a power law function (i.e., 1/*f*^*β*^). A lot of early studies on different types of signals labeled signals with 1/*f* power spectrums as noise (Keshner, 1982; Voytek et al., 2015; Zarahn et al., 1997), but more recent papers have argued that signals of interest (e.g., neural activity) can also have 1/*f* power spectrum (Freeman & Zhai, 2009; He, 2011, 2014; Podvalny et al., 2015). Signals with this kind of power spectrum are often called scale-free (He, 2011), which has been reported for many conditions (Ciuciu et al., 2012). For example, Churchill et al. reported that scale-free extents can be used for measuring cognitive effort (Churchill et al., 2016). We made two observations about the power law exponent of the results in Figure 12. First, based on both SSB+SWPC and SSB results, we can say that sensory domains (Vis, SM, and Temp) show power exponents that are closer to −1 for intradomain FNC compared to other inter- and intraconnections. He et al. reported a power law exponent closer to −1 for activity signals of the visual cortex and −0.6 for the motor cortex (He et al., 2010). Future works must explore the link between the estimated power law exponent of activity and connectivity time series, but based on previous works, we can say that the intra-FNC of the sensory functional domains shows higher complexity (Lee et al., 2021). Another observation we can make based on Figure 12 is that the power law exponent is more negative for SSB+SWPC compared to SWPC across many of the connections. The difference is significant for interfunctional connectivity power law exponent. As the power spectrum can be interpreted as the Fourier transform of the autocorrelation function (Wiener, 1930), the more negative exponent can be viewed as slower and weaker redundancy.

To compare the reoccurring trFNC patterns between the two FEP and TC groups, we used *k*-means to cluster the estimated trFNC matrices into four clusters. Then, we compared the dwell time between the two groups while controlling for nuisance regressors. After controlling for multiple comparisons, we found two clusters that showed significant results for SSB+SWPC (Figure 13) and one for SWPC (Figure 14). Using both methods, we can see that FEP individuals stay significantly more in cluster 1 compared to TC. By visually comparing the centroid for this cluster with other cluster centroids, we can say that cluster 1 shows an FNC pattern that is less connected compared to other centroids. This aligns with the dysconnectivity hypothesis of schizophrenia (Friston et al., 2016). Exclusive to SSB+SWPC result, we can see that TC individuals stay more in cluster 4 on average compared to FEP individuals. First, if we compare cluster 4 to cluster 1, we see a much stronger connection across all regions, which again aligns with the dysconnectivity hypothesis of schizophrenia. Additionally, cluster 4 shows strong negative FNC between subcortical and cortical regions. Similarly, Chen et al. reported decreased negative connectivity between many cortical regions and the thalamus and reported that these abnormal thalamic connectivities are related to the severity of clinical symptoms (Chen et al., 2019). Another paper reported abnormality in the functional connectivity between the hippocampus, posterior cingulate gyrus, and precuneus for adolescents with early-onset schizophrenia (Wen et al., 2021).

### Limitations

The major limitation of the proposed SSB+SWPC method is that it has an additional parameter compared with the classic SWPC method, namely the modulation frequency. The additional parameter gives us more freedom by essentially allowing us to design the low-pass filter of SWPC without worrying about the high-pass filter of the SWPC estimator. But this is a double-edged sword as additional parameters increase the complexity and points of failure of methods. For our specific added parameter, the user should make sure to select the modulation frequency in such a way that aliasing does not happen. Another limitation of the SSB+SWPC method is that it assumes that the signals are band-limited. As mentioned previously, we can probably overcome this limitation by up-sampling the time series before using SSB+SWPC. Still, such a method will introduce at least one additional parameter (i.e., up-sampling factor), which also increases the complexity of the method.

### Conclusion

Our proposed method uses single sideband frequency modulation to mitigate low-frequency information loss even if a small window size is used. We first describe the proposed method and then present several simulation scenarios to evaluate the performance of the proposed method in comparison with classic sliding window Pearson correlation (SWPC), under specific assumptions. Our simulation results showed that SSB+SWPC is significantly better than SWPC for estimating time-resolved connectivity. We also used the SSB+SWPC method to estimate trFNC from a real resting-state fMRI dataset to showcase its usage. Even in the real dataset, we showed that SSB+SWPC outperforms SWPC for the estimated time-resolved sample Pearson correlation. Using SSB+SWPC also resulted in significant differences between TC and FEP individuals, consistent with but extending prior work. Overall, the simulation and real fMRI results indicate that the proposed approach (i.e., SSB+SWPC) outperforms SWPC in many scenarios.

## SUPPORTING INFORMATION

Supporting information for this article is available at https://doi.org/10.1162/netn_a_00372. The function for estimating SSB+SWPC in both Python and MATLAB languages can be accessed through GitHub (https://github.com/trendscenter/SSBSWPC). We have also written a Jupyter Notebook to act as a demo for this paper and shared it on the GitHub page. Additionally, the function will also be implemented in the GIFT toolbox (https://trendscenter.org/software/). The data were not collected by us and are provided in a deidentified manner. The IRB will not allow sharing as the subjects did not sign a data reuse agreement during the original acquisition. We can share the derived results as they are not considered human subjects research.

## AUTHOR CONTRIBUTIONS

Ashkan Faghiri: Conceptualization; Formal analysis; Methodology; Software; Visualization; Writing – original draft. Kun Yang: Data curation; Writing – review & editing. Andreia Faria: Data curation. Koko Ishizuka: Data curation. Akira Sawa: Data curation. Tülay Adali: Writing – review & editing. Vince Calhoun: Conceptualization; Funding acquisition; Methodology; Resources; Supervision; Writing – review & editing.

## FUNDING INFORMATION

Vince Calhoun, National Science Foundation (https://dx.doi.org/10.13039/100000001), Award ID: 2112455. Vince Calhoun, National Institute of Mental Health (https://dx.doi.org/10.13039/100000025), Award ID: R01MH123610.

## Supplementary Material



## TECHNICAL TERMS

Independent component analysis: A method for decomposing multivariate signals into independent components.
Functional network connectivity: Temporal relationships between nodes that are networks themselves.
Time-resolved functional network connectivity: Relationships between nodes that change with time.
Sliding window Pearson correlation: An estimator that calculates sample Pearson correlation for small temporal windows instead of for the whole signal.
Modulation: The process of changing the frequency of a signal in this article.
Single sideband modulation: A method that modulates sidebands of the signal separately in the frequency domain that keeps the output real.
Analytic signal: A signal that only has non-negative frequency components.
Hilbert transform: A transform that shifts the phase of all frequency components of a signal by ±90 degrees.

## Supporting Information

https://github.com/trendscenter/SSBSWPC
