# Supplementary material for: Frequency modulation increases the specificity of time-resolved connectivity: A resting-state fMRI study
Source: Netw Neurosci. 2024 Oct 1;8(3):734–61. doi: 10.1162/netn_a_00372 (PMC11349031; doi:10.1162/netn_a_00372)
Supplement: Supplementary file 1 [file netn-8-3-734-s001.pdf]

Supplementary Materials

Frequency modulation increases the specificity of time-resolved connectivity: A resting-state fMRI study

Ashkan Faghiri[1], *Kun Yang[2], Andreia Faria[3], Koko Ishizuka[2], Akira Sawa[4]*, Tülay Adali[5], Vince Calhoun[1,6]

[1]Tri-Institutional Center for Translational Research in Neuroimaging and Data Science (TReNDS), Georgia State University, Georgia Institute of Technology, and Emory University, Atlanta, GA, USA.
[2]Department of Psychiatry, Johns Hopkins University School of Medicine, Baltimore, MD, USA.
[3]Department of Radiology and Radiological Science, Johns Hopkins University School of Medicine, Baltimore, MD 21205, USA.
[4]Johns Hopkins University School of Medicine and Bloomberg School of Public Health, Johns Hopkins Medical Institutions, Baltimore, MD, USA.
[5]University of Maryland, Baltimore County, Baltimore, MD, USA.
[6]School of Electrical and Computer Engineering, Georgia Institute of Technology, Atlanta, GA 30302, USA

Calculating the cutoff frequency for SWPC filters:

As pointed out in the manuscript, the sliding window Pearson correlation (SWPC) estimator can be broken down into three different sub-systems.

$$r_{x,y}(t;\Delta)=\sum_{\tau=t-\Delta}^{t+\Delta}\frac{(x(\tau)-\hat{\mu}_x(t))(y(\tau)-\hat{\mu}_y(t))}{\hat{\sigma}_x(t)\hat{\sigma}_y(t)} \quad \text{Eq1}$$

Here $\hat{\mu}_x(t)$ and $\hat{\sigma}_x(t)$ are the moving average and moving standard deviation for signal $x(t)$, estimated using the window function of SWPC. $2\Delta+1$ is the window length (in the number of samples) here.

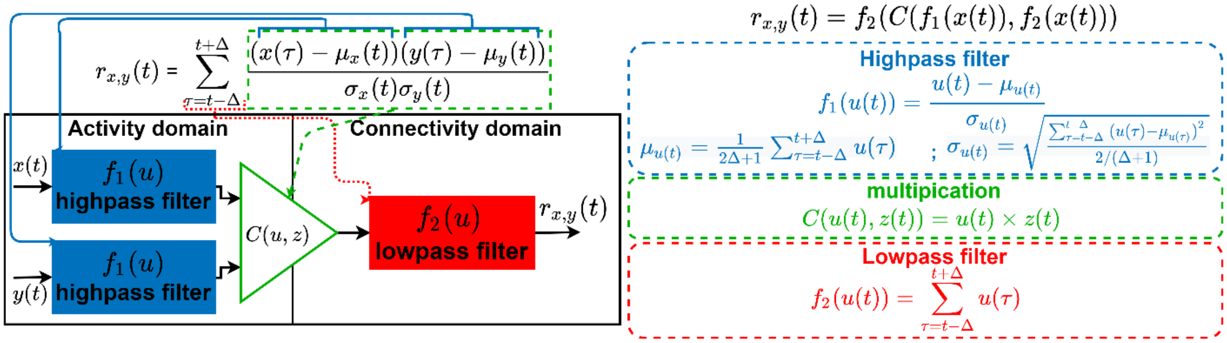

Figure 1. The system view of SWPC. Here, SWPC is broken into three different systems. The first systems (blue box) are high-pass filters that are applied to the activity signals (i.e., $x(t)$ and $y(t)$). We call the second system (green triangle) the coupling system, and it is the system that transforms the data from the activity domain to the connectivity domain. The third system (red box) applies a low-pass filter to the connectivity time series.

Figure 1 depicts the SWPC estimator as the combination of its sub-systems (Faghiri et al., 2022). As we can see here, SWPC consists of three sub-systems with their own functions (i.e., $f_1(u(t))$, $C(u(t),z(t))$, and $f_2(u(t))$). f_1 is the first sub-system in SWPC, and both signals (inputs of SWPC) pass through one instance of this function in parallel. Inside f_1, the moving average and moving standard deviation of the signal are calculated using the window function selected for SWPC, and the signal is normalized. Next, the output of

the two instances of f_1 are passed into the second sub-system of SWPC, i.e., $C(u(t), z(t)$ in Figure 1. This sub-system simply multiplies its two outputs. We call this a coupling function as this sub-system couples the information of the two signals together. Finally, the last sub-system is when the output of the coupling function is passed through a moving average function ($\sum_{\tau=n-\Delta}^{n+\Delta}\cdot$, i.e. $f_2(u(t))$ in Figure 1). Assume we have two domains: activity and connectivity. Function $f_1(u(t))$ modify the signals in the activity domain, while function $C(u(t), z(t))$ transfer the signals from the activity domain to the connectivity domains. Finally, $f_2(u(t))$ modify the signal in the connectivity domain.

In this section, we aim to mathematically show the impact of the first and third sub-systems of SWPC (i.e., $f_1(u(t))$ and $f_2(u(t))$) on the spectrum of their inputs. First, we want to point out that a moving average can be written a convolution of the signal with a window function. Assume we have a symmetric window function of length Δ:

$$h(n) = \frac{1}{2\Delta+1}\begin{cases}1 & -\Delta \le n \le \Delta \\ 0 & otherwise\end{cases} \qquad \text{Eq2}$$

We can use this to write a simple moving average (SMA) as a convolution:

$$\begin{aligned} SMA(x(n)) &= \frac{1}{2\Delta+1}\sum_{\tau=n-\Delta}^{n+\Delta} x(\tau) \\ &= \sum_{\tau=n-\Delta}^{n+\Delta} x(\tau)h(\tau) \\ &= \sum_{\tau=-\infty}^{\infty} x(\tau)h(\tau) \\ &= x(n) * h(n) \\ &= (2\Delta+1)f_2(x(t)) \end{aligned} \qquad \text{Eq3}$$

As we can see here, a moving average can be seen as a linear time-invariant system where the response function of the system is a low pass filter $h(n)$. Based on this, we can show that the first sub-system ($f_1(u(t))$ in Figure 1) is a high pass filter: Ignoring the denominator in this sub-system (i.e., $\frac{(x(n)-\hat{\mu}_x(n))}{\hat{\sigma}_x(n)}$) we can write:

$$\begin{aligned} f_1(x(t)) = x(n) - \hat{\mu}_x(n) &= x(n) - x(n) * h(n) \\ &= x(n) * (\delta(n) - h(n)) \end{aligned} \qquad \text{Eq4}$$

Where $\delta(n)$ is the delta function. It is trivial to show that $\delta(n) - h(n)$ is a high pass filter if $h(n)$ is a low-pass filter. So, as mentioned before, the first sub-system of SWPC is a high pass filter, while the last sub-system ($f_2(u(t))$ in Figure 1) is a low-pass filter.

If we want to examine the impact of SWPC on the spectrum of the inputs (i.e., $x(n)$ and $y(n)$) We first have to derive the discrete Fourier transform (DFT) of the window function used in SWPC (i.e., $h(n)$) as it impacts both the first and third sub-systems of SWPC based on Eq3 and Eq4. Here, we assume the window is a rectangular window, but similar proofs can be extended to other windows, such as Gaussian. For simplicity's sake, we try to first derive the discrete Fourier transform (DTFT) and then find DFT based on that:

Supplementary Materials

The equation for a rectangular window with length M is:

$$h_{rect}(n) = \frac{1}{M}\begin{cases} 1 & -\frac{M-1}{2} \le n \le \frac{M-1}{2} \\ 0 & otherwise \end{cases} \qquad \text{Eq5}$$

Note that here, we used M instead of $2\Delta + 1$ (as done above) for simplicity. One can replace these two easily in either equation. The DTFT of $w_{rect}(n)$ is then:

$$\begin{aligned} H_{rect}(\omega) &\overset{\text{def}}{=} \sum_{n=-\infty}^{\infty} h_{rect}(n)\, e^{-j\omega n} \qquad \omega \in [-\pi, \quad \pi) \\ &= \frac{1}{M}\sum_{n=-\frac{M-1}{2}}^{\frac{M-1}{2}} e^{-j\omega n} = \frac{1}{M}\frac{e^{j\omega\frac{M-1}{2}} - e^{-j\omega\frac{M+1}{2}}}{1 - e^{-j\omega}} \end{aligned} \qquad \text{Eq6}$$

The last line was derived using the closed form of a geometric series (see proof in Eq9). We can simplify this further:

$$\begin{aligned} H_{rect}(\omega) &= \frac{1}{M}\frac{e^{-\frac{j\omega}{2}}}{e^{-\frac{j\omega}{2}}}\left(\frac{e^{\frac{j\omega M}{2}} - e^{-\frac{j\omega M}{2}}}{e^{\frac{j\omega}{2}} - e^{-\frac{j\omega}{2}}}\right) \\ &= \frac{1}{M}\frac{\sin\left(\frac{\omega M}{2}\right)}{\sin\left(\frac{\omega}{2}\right)} \end{aligned} \qquad \text{Eq7}$$

Which is equal to the aliased sinc function (also called the Dirichlet function or *periodic sinc*). Note that $H_{rect}(\omega)$ in Eq7 is the DTFT of $h_{rect}(n)$. To get the DFT, we can simply replace ω with $\frac{2\pi}{N}k$ where N is the length of the time series, and k is the index of frequency. The transfer function of the first sub-system ($f_1(u(t))$ in Figure 1) is then $1 - H_{rect}(\omega)$ which is a highpass filter.

The transfer function of the last sub-system is $H_{rect}(\omega)$ and thus is a low-pass filter. It is typical to describe filters in terms of their cut-off frequencies. Here, we derive the -3db (decibel) attenuation for the cut-off frequencies because it corresponds to approximately half the power of the filters. This attenuation is equal to $\sqrt{0.5} \approx 0.707$ fall in the amplitude of the transfer function. $H_{rect}(\omega) = \sqrt{0.5}$ does not have a symbolic solution. We can solve this equation by approximating $H_{rect}(\omega)$ with its Taylor series. This is not a trivial derivation, but there have been some attempts to solve this problem[1,2,3]. All in all, we can estimate the -3db cutoff for $H_{rect}(\omega)$ using this approximation, which results in $f_{LPF-cutoff}(\omega) \approx \frac{0.44}{\sqrt{M^2-1}}F_S$, where M is the size of the window, and F_s is the sampling frequency. For the highpass filter (i.e., $\delta(n) - h(n)$) we can approximate the cutoff frequency as twice the cutoff frequency of the low pass filter ($h(n)$); $f_{HPF-cutoff}(\omega) \approx \frac{0.88}{\sqrt{M^2-1}}F_S$. Note that all these values are approximations and are more accurate for rectangular windows. Having mentioned that our values are more accurate compared to previous studies where instead of 0.88 (in $f_{HPF-cutoff}(\omega)$ numerator), the value is mentioned to be 1 (Leonardi & Van De Ville, 2015). In Figure 3 of our manuscript, the optimal window size is selected as the largest window size

[1] Power Series Expansions of the Dirichlet Function (https://www.mers.byu.edu/docs/reports/MERS1901.pdf)
[2] https://dsp.stackexchange.com/questions/9966/what-is-the-cut-off-frequency-of-a-moving-average-filter
[3] https://dsp.stackexchange.com/questions/28169/3db-cut-off-frequency-of-moving-average

that allows us to capture connectivity with the frequency of f_{corr} based on the cutoff frequency of the low pass filter of SWPC ($f_{LPF-cutoff}(\omega) \approx \frac{0.44}{\sqrt{M^2-1}} F_S$)

Closed form of geometric series

The closed form of geometric series, which is defined as the sum of a geometric sequence $S(x) = \sum_{n=L}^{U} x^n$ is:

$$S(x) = \frac{x^L - x^{U+1}}{1-x} \quad \text{Eq8}$$

Proof:

$$\begin{aligned} S(x) &= x^L + x^{L+1} + x^{L+2} + x^{L+3} + \cdots + x^U \\ xS(x) &= x^{L+1} + x^{L+2} + x^{L+3} + x^{L+4} + \cdots + x^{U+1} \\ \Rightarrow S(x) - xS(x) &= x^L - x^{U+1} \\ \Rightarrow S(x) &= \frac{x^L - x^{U+1}}{1-x} \end{aligned} \quad \text{Eq9}$$

Elbow Method for cluster number selection:

For selecting the number of clusters (or states) using the k-means clustering, we used the elbow method. In this method, we looked at the average within-cluster distance and selected the elbow point as a good cluster number. This method is usually a visual method, but in this work, we have tried to make it more replicable. We have essentially fitted two lines to the data. One to the start of the within-cluster distance vector and one to its end. Then, the point that these two lines cross is assumed to be the elbow. Figure 2 shows the results of the elbow method.

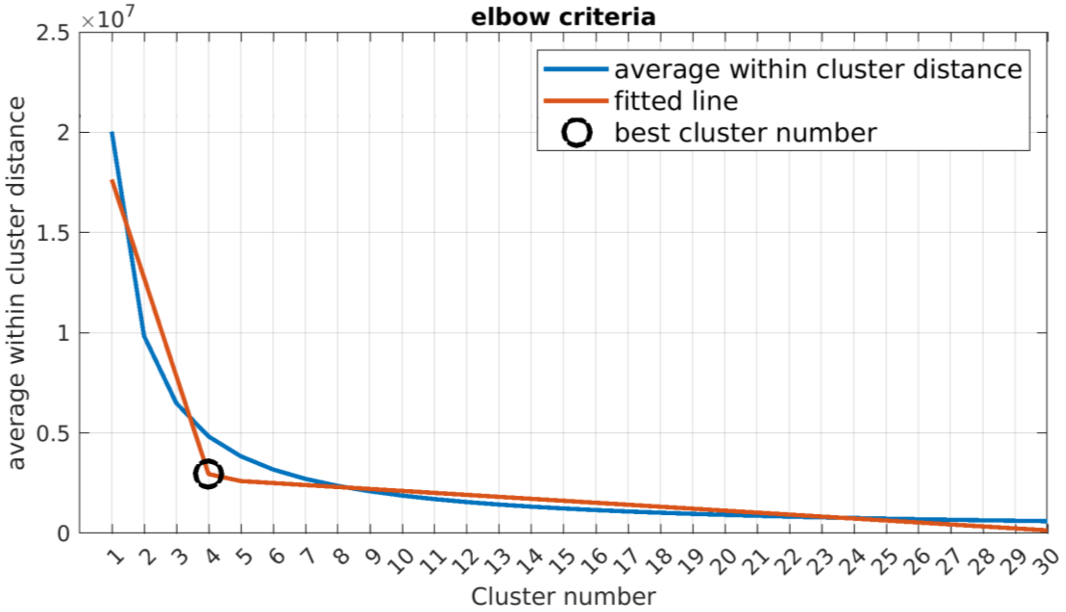

Figure 2. Results of the elbow method used for selecting the best cluster number.

Supplementary Materials

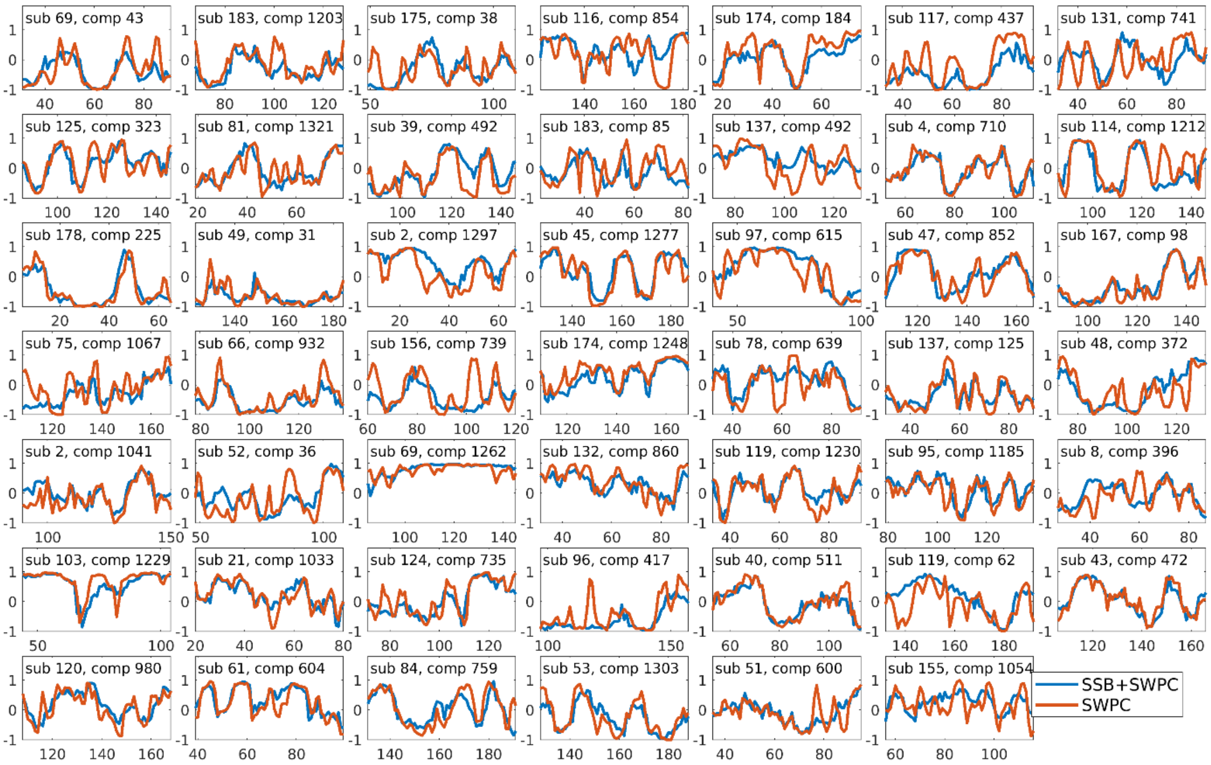

Figure 3. This figure shows samples of estimated trFNC using both SSB+SWPC and SWPC methods for several random individuals and component pairs. At first glance, it seems like SSB+SWPC results in less rapid jumps in the estimation. This can point to the fact that SSB+SWPC is possibly less "noisy". Of course, as we do not know the ground truth, we can not say for sure if these rapid jumps are actually noise, but in most cases, we assume that rapid jumps in signals are not desirable.

References

Faghiri, A., Iraji, A., Duda, M., Adali, T., & Calhoun, V. D. (2022). A Unified Framework for Modularizing and Comparing Time-Resolved Functional Connectivity Methods. 2022 44th Annual International Conference of the IEEE Engineering in Medicine & Biology Society (EMBC),

Leonardi, N., & Van De Ville, D. (2015). On spurious and real fluctuations of dynamic functional connectivity during rest. *Neuroimage*, *104*, 430-436.
